# Evaluation of an automated laminar cartilage T2 relaxation time analysis method in an early osteoarthritis model

**DOI:** 10.1007/s00256-024-04786-1

**Published:** 2024-09-04

**Authors:** Wolfgang Wirth, Susanne Maschek, Anna Wisser, Jana Eder, Christian F. Baumgartner, Akshay Chaudhari, Francis Berenbaum, Felix Eckstein, on behalf of the OA-BIO Consortium

**Affiliations:** 1 Chondrometrics GmbH, Freilassing, Germany; 2 Research Program for Musculoskeletal Imaging, Institute of Imaging & Functional Musculoskeletal Research, Center of Anatomy & Cell Biology, Paracelsus Medical University, Strubergasse 21, 5020 Salzburg, Austria; 3 Ludwig Boltzmann Inst. for Arthritis and Rehabilitation (LBIAR), Paracelsus Medical University, Strubergasse 21, 5020 Salzburg, Austria; 4 University of Tübingen, Tübingen, Germany; 5 Department of Radiology, Stanford University, Stanford, CA USA; 6 4Moving Biotech, Lille, France; 7 Department of Rheumatology, AP-HP Saint-Antoine Hospital, Paris, France; 8 Sorbonne University, AP-HP Saint-Antoine Hospital, INSERM, Paris, France

**Keywords:** Osteoarthritis, Cartilage T2, Knee, MRI, Deep learning, U-Net

## Abstract

**Objective:**

A fully automated laminar cartilage composition (MRI-based T2) analysis method was technically and clinically validated by comparing radiographically normal knees with (CL-JSN) and without contra-lateral joint space narrowing or other signs of radiographic osteoarthritis (OA, CL-noROA).

**Materials and methods:**

2D U-Nets were trained from manually segmented femorotibial cartilages (*n* = 72) from all 7 echoes (All_E_), or from the 1st echo only (1^st^_E_) of multi-echo-spin-echo (MESE) MRIs acquired by the Osteoarthritis Initiative (OAI). Because of its greater accuracy, only the All_E_ U-Net was then applied to knees from the OAI healthy reference cohort (*n* = 10), CL-JSN (*n* = 39), and (1:1) matched CL-noROA knees (*n* = 39) that all had manual expert segmentation, and to 982 non-matched CL-noROA knees without expert segmentation.

**Results:**

The agreement (Dice similarity coefficient) between automated vs. manual expert cartilage segmentation was between 0.82 ± 0.05/0.79 ± 0.06 (All_E_/1^st^_E)_ and 0.88 ± 0.03/0.88 ± 0.03 (All_E_/1^st^_E_) across femorotibial cartilage plates. The deviation between automated vs. manually derived laminar T2 reached up to − 2.2 ± 2.6 ms/ + 4.1 ± 10.2 ms (All_E_/1^st^_E_). The All_E_ U-Net showed a similar sensitivity to cross-sectional laminar T2 differences between CL-JSN and CL-noROA knees in the matched (Cohen’s *D* ≤ 0.54) and the non-matched (*D* ≤ 0.54) comparison as the matched manual analyses (*D* ≤ 0.48). Longitudinally, the All_E_ U-Net also showed a similar sensitivity to CL-JSN vs. CS-noROA differences in the matched (*D* ≤ 0.51) and the non-matched (*D* ≤ 0.43) comparison as matched manual analyses (*D* ≤ 0.41).

**Conclusion:**

The fully automated T2 analysis showed a high agreement, acceptable accuracy, and similar sensitivity to cross-sectional and longitudinal laminar T2 differences in an early OA model, compared with manual expert analysis.

**Trial registration:**

Clinicaltrials.gov identification: NCT00080171.

**Supplementary Information:**

The online version contains supplementary material available at 10.1007/s00256-024-04786-1.

## Introduction

Osteoarthritis (OA) is a chronic disease and a leading cause of disability [1, 2]. The knee is the most frequently affected joint [3] and in the absence of knee-specific risk factors such as traumatic injuries, knee OA frequently presents as a bilateral disease [4, 5]. A previously established early OA model demonstrated that radiographically normal knees (Kellgren & Lawrence grade [KLG] 0) with contralateral radiographic joint space narrowing (JSN, CL-JSN knees) exhibit greater cartilage thickness loss than such knees without contralateral JSN or other signs of radiographic OA (CL-noROA knees) [6].

Cartilage transverse relaxation time (T2) is sensitive to differences in cartilage composition between knees with and without OA [7], due to alterations in hydration and collagen integrity and orientation [8, 9]. In the above early OA model, the CL-JSN knees showed a prolonged superficial layer T2 at baseline and a greater increase in deep layer T2 over 3 years than CL-noROA knees [10]. Radiographically normal knees with unilateral (contra-lateral) JSN may thus permit studying knee OA at a very early stage, before radiographic alterations and loss of cartilage substance occur. Further, they represent an ideal model for clinically validating novel approaches of automated cartilage T2 analyses, by comparing differences between CL-JSN and CL-noROA knees to those observed using manual expert analyses.

Deep learning (DL) and in particular convolutional neural networks (CNN) have been established for segmenting articular cartilage morphology (volume/ thickness) from high-resolution MRIs: Recent studies reported high agreement with reference segmentations [11–13], and similar sensitivity to differences in longitudinal cartilage loss between knees with different progression profiles [14]. Only few studies thus far used CNNs for segmenting articular cartilage from multi-echo spin-echo (MESE) MRIs that support cartilage T2 analysis [15–17]. Two of these studies focused on technical validation only [15, 16]. Razmjoo et al. investigated the association of cartilage T2 with demographic variables, pain, and KL grade, and reported T2 to be associated with the development of incident radiographic OA as well as subsequent knee replacement surgery [17]. Yet, no previous study compared the sensitivity of fully automated cartilage T2 analyses to between-group differences in laminar T2 with that of manual expert cartilage T2 analyses. Such a comparison is, however, needed before an automated T2 analysis technique can be applied to clinical trials or large-scale research studies.

The objective of this study was thus to technically and clinically validate the performance of a U-Net-based CNN cartilage T2 analysis:

- For technical validation, we evaluated the agreement and differences in laminar cartilage T2 between U-Net-based and manual expert cartilage segmentation and evaluated the performance of U-Nets trained on all 7 MESE echoes vs. U-Nets trained on the 1st echo only.
- For clinical validation, we relied on a previously published early OA model [10] in order to compare the sensitivity of U-Net-based T2 analysis to cross-sectional and longitudinal differences in laminar cartilage T2 between CL-JSN knees and CL-noROA knees with the sensitivity observed using manual expert analysis in a matched and a larger non-matched analysis.

## Materials and methods

### Early OA model

The Osteoarthritis Initiative (OAI) enrolled 4796 participants. Of these, 3284 had risk factors for developing symptomatic and/or radiographic knee OA (incidence cohort), whereas 122 did neither have such risk factors nor signs or symptoms of knee OA (healthy reference cohort = HRC) [18]. The OAI (clinicaltrials.gov: NCT00080171, https://data-archive.nimh.nih.gov/oai/) was approved by the Committee on Human Research, the Institutional Review Board of the University of California, San Francisco (UCSF) and the institutional review boards of all clinical sites. All OAI participants provided written informed consent. The current study was carried out in accordance with the OAI data user agreement and both the OAI and the current study were performed in accordance with the ethical standards of the institutional research committees and with the 1964 Helsinki Declaration and its later amendments.

**Fig. 1:**
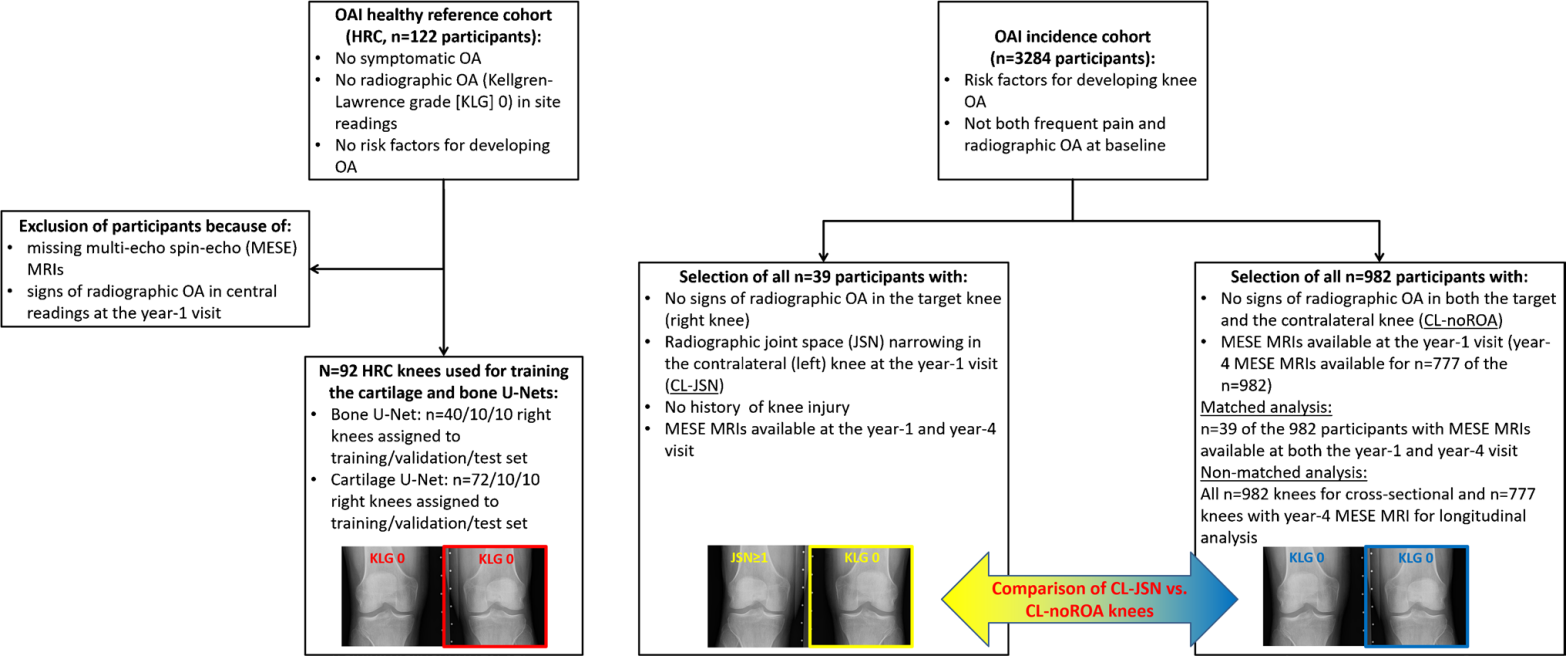
Illustration of the selection of participants and of the design of the clinical validation study comparing laminar cartilage T2 between radiographically normal knees with contralateral joint space narrowing (CL-JSN) and radiographically normal knees without signs of radiographic OA (ROA) in the contralateral knee (CL-noROA)

Our study included knees without signs of radiographic OA from OAI incidence cohort and HRC participants. Participants from the HRC were used for training the U-Nets for the automated segmentation, whereas the clinical validation relied on a subcohort of knees from the OAI incidence cohort. Figure 1 illustrates the selection of participants and the design of the clinical validation study. The subcohort used for the clinical validation was specifically selected for studying the impact of contralateral OA status on cartilage thickness and T2 (change) between year 1 and 4 [6, 10] in radiographically normal knees without a history of knee injury. The study focused on the 39 right KLG0 knees with contralateral JSN, as MESE MRIs were only acquired in right knees in the OAI. These 39 CL-JSN knees were matched 1:1 to 39 right CL-noROA knees without contralateral JSN, and without other signs of radiographic OA in the ipsi- or contralateral knee (criteria: same sex and pain frequency, similar age [± 5 y] and BMI [± 5 kg/m^2^]).

To evaluate whether automated analysis can be scaled to a large cohort and to explore how this affects the comparison between CL-JSN and CL-noROA knees (non-matched analysis), we additionally conducted a non-matched comparison between the 39 CL-JSN knees and all bilaterally normal OAI participants with MESEs MRIs available. The OAI incidence cohort comprised 1024 eligible participants with bilateral KLG0, of which 42 had to be excluded because the Y1 MRIs were missing. Another 205 knees had to be excluded from the longitudinal analysis because the Y4 MRIs were missing. The non-matched group of CL-noROA knees therefore comprised 982 knees for the cross-sectional analysis and 777 for the longitudinal analysis. Of these, 39 knees were also part of the matched group of CL-noROA knees. Participants from the HRC were excluded from this comparison because cartilage T2 was previously reported to be significantly shorter in these participants [19] and because the U-Nets were trained using those HRC knees.

### Imaging

**Fig. 2:**
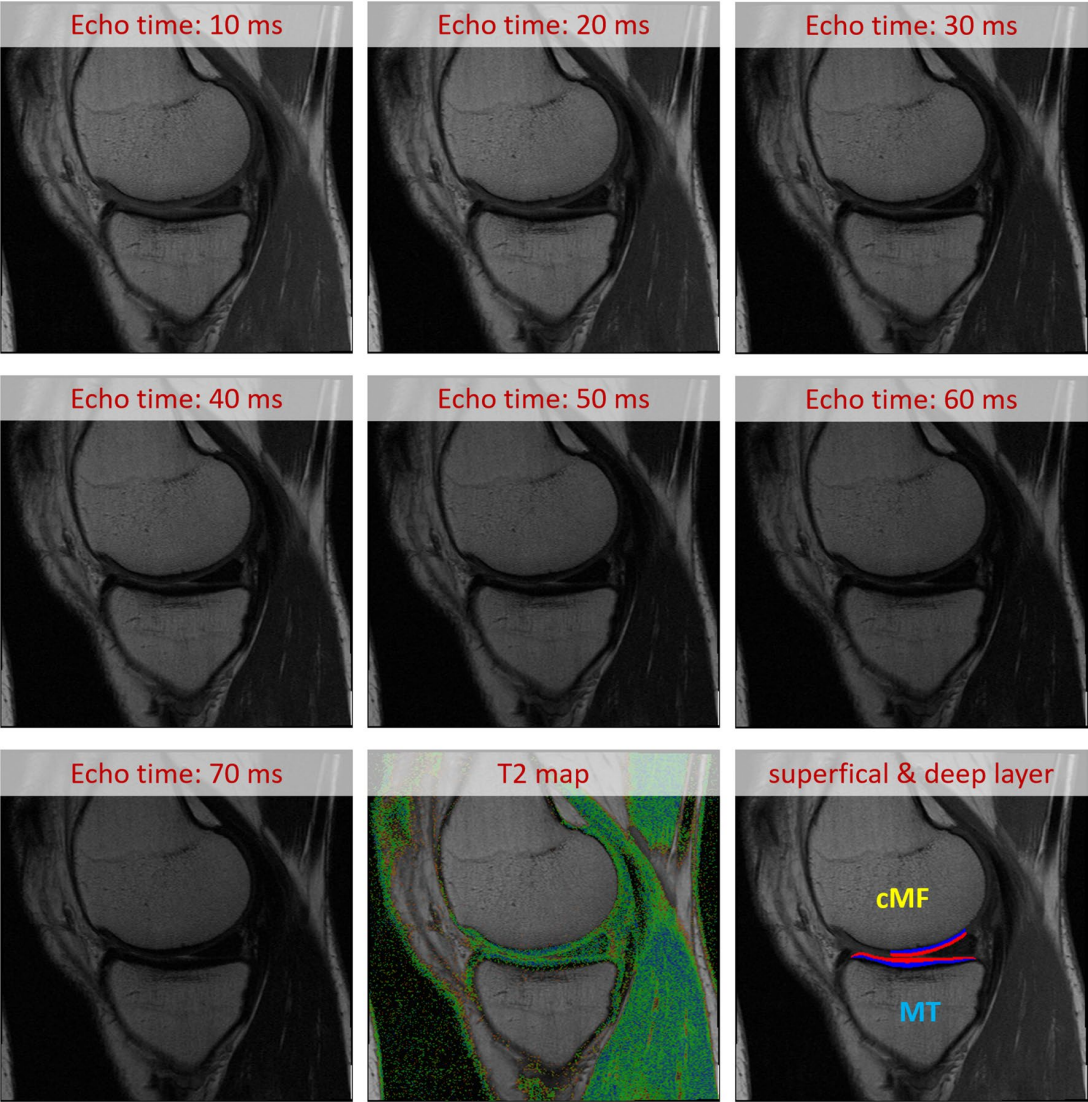
Illustration of the multi-echo spin-echo (MESE) MRIs acquired by the osteoarthritis initiative (OAI). The figure shows the 7 echoes that were acquired with echo times between 10 and 70 ms, the T2 map computed from these 7 echoes, and the separation of the femorotibial cartilages into the superficial (red) and deep (blue) cartilage layer

MRIs were acquired using 3 T Magnetom Trio scanners (Siemens Healthineers, Erlangen, Germany) and quadrature transmit/receive knee coils (USA Instruments, Aurora, OH). Sagittal MESE MRIs were obtained with 3 mm slice thickness (0.5 mm slice gap), 0.31 mm in-plane resolution, 2700 ms repetition time, and 10, 20, 30, 40, 50, 60, and 70 ms echo times (Fig. 2) [20].

### Cartilage and bone segmentation

Manual segmentation of the femorotibial cartilages was performed for the matched CL-JSN/CL-noROA pairs (*n* = 39) by an expert reader (SM) who traced the cartilage surfaces and the bone interfaces of the medial and lateral tibia (MT/LT), and of the central (weight-bearing) medial and lateral femoral condyle (cMF/cLF) in each sagittal slice [10]. The year-1 and -4 MRIs were analyzed with reference to each other, with blinding to acquisition order and contralateral ROA status.

**Fig. 3:**
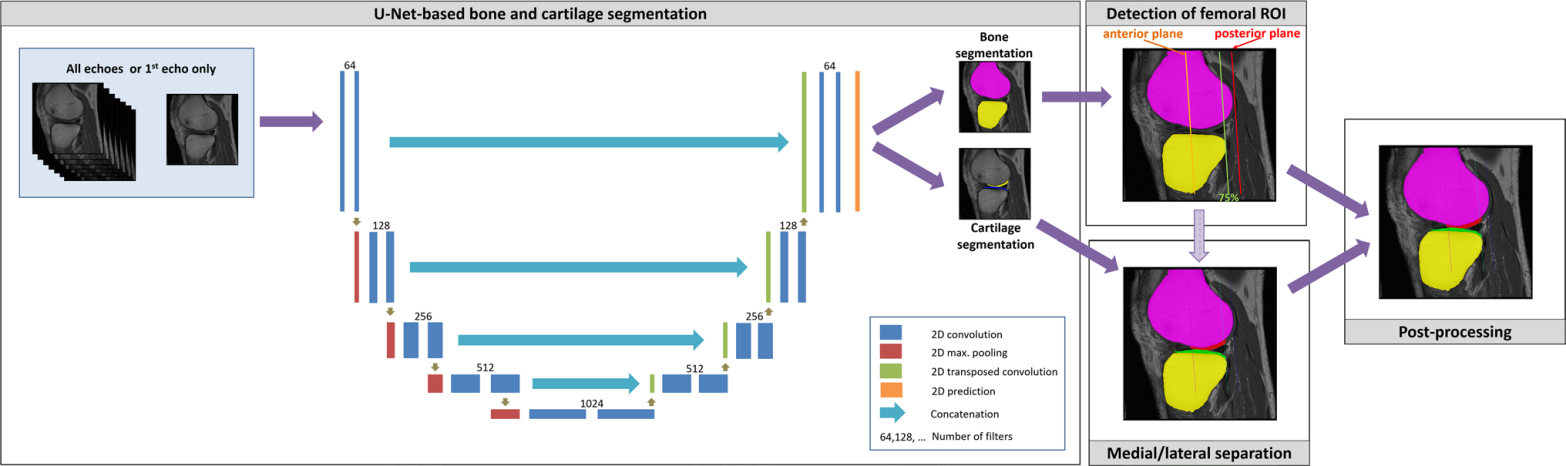
Illustration of the workflow used for the fully automated segmentation of femorotibial cartilages. The workflow relied on a 2D U-Net segmentation of bones and a 2D U-Net segmentation of the femorotibial cartilages. Both U-Nets were trained using either all available MESE MRI echoes or the 1st MESE MRI echo only. After the U-Net-based segmentation, the femoral region of interest was computed from the bone segmentations and the cartilage segmentations were separated into medial and lateral compartment segmentations using the femoral segmentation as landmark. As last step, an automated post-processing was performed in order to clean the segmentations

The automated segmentation of the femorotibial cartilages from the MESE MRIs relied on a 2D U-Net-based segmentation of the tibial, fibular, and femoral bones that provided anatomical landmarks, and a U-Net-based cartilage segmentation for evaluating deep and superficial T2 (Fig. 3) [21]. The training of both the bone and the cartilage U-Nets was performed once using all 7 echoes of the MESE (“All_E_” U-Net) and once using the 1st echo only (“1^st^_E_” U-Net) that had an echo time of 10 ms. In order to take the multiple MESE echoes into account, the first convolutional layer of the All_E_ U-Net comprised 7 input channels whereas the first convolutional layer of the 1^st^_E_ U-Net comprised 1 input channel only.

The bone segmentation U-Nets were trained using 60 MESE MRIs from the OAI HRC (training/validation/test set: *n* = 40/10/10), with manual segmentations of the distal femur, proximal tibia, and proximal fibula performed by experienced readers. The cartilage segmentation U-Nets were trained using MESE MRIs from the 92 OAI HRC participants who were confirmed to be free of radiographic signs of OA in central radiographic readings (training/validation/test set: *n* = 72/10/10). The manual segmentation of the femorotibial cartilages was again performed by experienced readers [19, 22]. The knees assigned to the validation/test sets were identical for bone and cartilage U-Nets.

The U-Nets were trained on all MRI slices, using a weighted cross entropy loss function and Adam optimization with an initial learning rate of 0.01. All network weights were randomly initialized using the TensorFlow variance scaling initializer. The software was implemented in Python (Python Software Foundation, DE, USA) using the TensorFlow framework (Google LLC, Mountain View, CA, USA), and was extended from software previously used for morphometric analysis of femorotibial cartilage from high-resolution 3D MRI [13]. The bone U-Nets were trained using separate labels for the distal femur (weight: 0.25), proximal tibia (weight: 0.25), and proximal fibula (weight: 0.3, background weight: 0.2). The cartilage U-Nets were trained using 2 labels, one for the tibial (medial/lateral, weight 0.4) and one for the central femoral cartilage (medial/lateral, weight 0.4, background weight: 0.2). The label weights have been determined empirically. A mini-batch size of 4 slices per training iteration was used for training the model over a total of 50 epochs (without early stopping). The parameter set of the iteration that provided the best Dice agreement with manual segmentations in the validation set was selected. The training required approximately 2.5 h for each of the bone U-Nets and approximately 5 h for each of the cartilage U-Nets using an NVIDIA GeForce RTX 2080 Ti GPU. The landmarks required for distinguishing medial and lateral compartment cartilage, and for determining the central part of the femoral cartilages were derived automatically from the U-Net-based bone segmentations (Supplement 1 and Supplemental Fig. 1).

The automated U-Net-based cartilage segmentation was subsequently applied to three datasets: the 10 HRC test set knees, the 2 × 39 matched pairs, the 943 year-1 and 777 year-4 scans of the non-matched CL-noROA knees (*n* = 1847 scans in total: 10 HRC, 39 × 2 year-1 and year-4 scans of CL-JSN knees, 982 year-1 and 777 year-4 scans of CL-noROA knees). The analysis took approximately 40 s for each MESE volume (5 s for the U-Net and 35 s for post-processing). In the next step, the U-Net training was repeated with a different assignment of knees into training, validation, and test sets in order to evaluate the consistency of the results. These U-Nets were again applied to 10 (other) knees from the HRC test set and the 2 × 39 matched pairs. No manual quality control or corrections, whatsoever, were performed for the automatically segmented bones and cartilages. Instead, an automated post-processing step was implemented to address various issues, such as filling small gaps by detecting enclosed unsegmented areas, eliminating implausible segmentations (e.g., fragments not connected to the main segmentation in the same or other slices), and smoothing of segmentation spikes.

### Cartilage T2 analysis

Cartilage T2 was computed for each segmented voxel by fitting a mono-exponential decay curve to the measured signal intensities [19]. Because T2 varies with tissue depth [8], the cartilage plates were divided into the top (superficial) and bottom (deep) 50%, based on the distance of each voxel between the cartilage surface and bone interface (Fig. 2) [23].

### Statistical analysis

All analyses were performed using SPSS 27 (IBM Corporation, Armonk, NY, USA).

#### Agreement between automated vs. manual bone and cartilage segmentations

The agreement between U-Net-based and manual cartilage segmentations was assessed across 88 knees with manual cartilage segmentations available (10 HRC, 39 CL-JSN, 39 CL-noROA), the agreement between U-Net-based and manual bone segmentations was assessed across 10 HRC knees with manual bone segmentations available. The evaluation was conducted using several metrics, including the Dice similarity coefficient (DSC), the Hausdorff distance (HD), the average symmetric surface distance (ASSD), and the volume overlap error (VOE). Agreement was rated as high if the DSC was 0.80 or greater.

#### Comparison of laminar T2 between U-Net and manual expert analysis

Laminar cartilage T2 was compared between U-Net-based and manual segmentations using paired *t*-tests, Pearson correlation coefficients, and Bland–Altman plots in the same 88 knees. These analyses were repeated for the re-trained U-Nets.

#### Cross-sectional and longitudinal comparison between CL-JSN and CL-noROA knees

Differences in baseline laminar cartilage T2, and in 3-year change in T2 were compared between CL-JSN and CL-noROA knees using paired *t*-tests and Cohen’s *D* as measure of effect size (paired analysis). These analyses focused on the total femorotibial joint (FTJ), the medial (MFTC), and the lateral femorotibial compartment (LFTC), whereas results for individual cartilage plates are shown in Supplemental Tables 6 and 7. These comparisons were performed in the 1:1 matched sample to directly compare the sensitivity to contralateral ROA status of the automated U-Net-based analysis with that of the manual expert analysis. In addition, a non-matched comparison was performed using the full sample of eligible CL-noROA knees (unpaired analysis), to evaluate the applicability and robustness of the automated analysis pipeline in a large sample, and to determine whether the results were consistent with those from the matched comparison. These non-matched comparisons were performed using unpaired *t*-tests without adjustment for demographic covariates, since laminar cartilage T2 has been observed to not be dependent on age and sex in knees without radiographic OA [22]. Because the 1^st^_E_ U-Net showed a lower accuracy than the 1^st^_E_ U-Net for superficial layer T2, these comparisons were only performed for the manual expert and the All_E_ U-Net analysis.

## Results

All 1847 available scans (Fig. 1) were successfully analyzed using the automated, U-Net-based segmentation method without any manual correction required. Demographic data for all participants are reported in Supplemental Table 1.

### Agreement between automated vs. manual bone and cartilage segmentations

The agreement between U-Net-based and manual bone and cartilage segmentations is shown in Table 1. For bone segmentations, a high agreement was observed between the automated and manual approaches for both U-Nets. The DSC amounted to 0.98 ± 0.00 (both All_E_ and 1^st^_E_) for both the femur and tibia and to 0.93 ± 0.03/0.92 ± 0.03 (All_E_/1^st^_E_) for the fibula.

**Table 1:** Agreement between U-Net-based and manual bone and cartilage segmentations from multi-echo spin-echo MRIs and comparison of the agreement between the U-Net trained from all echoes (All_E_) and the U-Net trained from the 1st echo (1^st^_E_) in all knees with manual segmentations

		All_E_ U-Net		1^st^_E_ U-Net		All_E_ vs. 1^st^_E_ U-Net		
		Mean ± SD	[95% CI]	Mean ± SD	[95% CI]	Diff. ± SD	[95% CI]	*p*-value
Bone (HRC test set, *n* = 10)								
DSC	**Femur**	0.98 ± 0.00	[0.98, 0.98]	0.98 ± 0.00	[0.98, 0.98]	0.00 ± 0.00	[0.00, 0.00]	0.01
	**Tibia**	0.98 ± 0.00	[0.98, 0.98]	0.98 ± 0.00	[0.98, 0.98]	0.00 ± 0.00	[0.00, 0.00]	0.02
	**Fibula**	0.93 ± 0.03	[0.91, 0.96]	0.92 ± 0.03	[0.91, 0.94]	0.01 ± 0.01	[0.00, 0.02]	0.01
HD (mm)	**emur**	4.12 ± 1.30	[3.19, 5.05]	4.90 ± 2.77	[2.92, 6.88]	− 0.78 ± 1.64	[− 1.96, 0.39]	0.17
	**Tibia**	3.87 ± 0.52	[3.50, 4.25]	4.98 ± 2.15	[3.44, 6.52]	− 1.11 ± 2.23	[− 2.70, 0.49]	0.15
	**Fibula**	5.54 ± 3.63	[2.94, 8.14]	5.39 ± 3.13	[3.15, 7.63]	0.15 ± 2.20	[− 1.43, 1.72]	0.84
ASSD (mm)	**Femur**	0.17 ± 0.05	[0.14, 0.21]	0.21 ± 0.09	[0.15, 0.28]	− 0.04 ± 0.05	[− 0.08, 0.00]	0.04
	**Tibia**	0.15 ± 0.05	[0.11, 0.18]	0.20 ± 0.09	[0.14, 0.26]	− 0.05 ± 0.07	[− 0.10, 0.00]	0.03
	**Fibula**	0.45 ± 0.40	[0.16, 0.73]	0.50 ± 0.36	[0.24, 0.76]	− 0.05 ± 0.10	[− 0.13, 0.02]	0.11
VOE (%)	**Femur**	0.04 ± 0.01	[0.03, 0.04]	0.04 ± 0.01	[0.03, 0.05]	0.00 ± 0.00	[− 0.01, 0.00]	0.01
	**Tibia**	0.03 ± 0.00	[0.03, 0.04]	0.04 ± 0.01	[0.03, 0.04]	0.00 ± 0.00	[− 0.01, 0.00]	0.02
	**Fibula**	0.12 ± 0.05	[0.09, 0.16]	0.14 ± 0.04	[0.11, 0.17]	− 0.02 ± 0.02	[− 0.03, − 0.01]	0.01
Cartilage (HRC test set, matched CL-JSN and CL-noROA knees, *n* = 88)								
DSC	**MT**	0.87 ± 0.03	[0.87, 0.88]	0.86 ± 0.03	[0.86, 0.87]	0.01 ± 0.02	[0.01, 0.02]	< 0.01
	**cMF**	0.82 ± 0.05	[0.81, 0.83]	0.79 ± 0.06	[0.78, 0.80]	0.03 ± 0.05	[0.01, 0.04]	< 0.01
	**LT**	0.88 ± 0.03	[0.88, 0.89]	0.88 ± 0.03	[0.87, 0.89]	0.00 ± 0.02	[0.00, 0.01]	0.08
	**cLF**	0.83 ± 0.04	[0.82, 0.84]	0.82 ± 0.05	[0.81, 0.83]	0.01 ± 0.04	[0.00, 0.02]	0.03
HD (mm)	**MT**	4.12 ± 1.48	[3.80, 4.43]	4.12 ± 1.10	[3.89, 4.35]	− 0.01 ± 1.30	[− 0.28, 0.27]	0.97
	**cMF**	4.66 ± 1.80	[4.28, 5.04]	4.59 ± 1.67	[4.24, 4.95]	0.06 ± 1.49	[− 0.25, 0.38]	0.69
	**LT**	4.48 ± 3.69	[3.70, 5.26]	4.23 ± 3.71	[3.44, 5.02]	0.25 ± 1.36	[− 0.04, 0.54]	0.09
	**cLF**	4.34 ± 1.64	[3.99, 4.69]	4.41 ± 1.56	[4.08, 4.74]	− 0.07 ± 2.00	[− 0.49, 0.36]	0.75
ASSD (mm)	**MT**	0.18 ± 0.12	[0.15, 0.20]	0.20 ± 0.13	[0.18, 0.23]	− 0.03 ± 0.09	[− 0.05, − 0.01]	0.01
	**cMF**	0.33 ± 0.24	[0.28, 0.38]	0.40 ± 0.26	[0.35, 0.46]	− 0.07 ± 0.20	[− 0.11, − 0.03]	< 0.01
	**LT**	0.19 ± 0.09	[0.17, 0.21]	0.19 ± 0.10	[0.16, 0.21]	0.01 ± 0.09	[− 0.01, 0.03]	0.49
	**cLF**	0.23 ± 0.17	[0.19, 0.27]	0.25 ± 0.19	[0.21, 0.30]	− 0.03 ± 0.23	[− 0.07, 0.02]	0.28
VOE (%)	**MT**	0.22 ± 0.04	[0.21, 0.23]	0.24 ± 0.04	[0.23, 0.25]	− 0.02 ± 0.03	[− 0.02, − 0.01]	< 0.01
	**cMF**	0.31 ± 0.06	[0.30, 0.32]	0.34 ± 0.08	[0.32, 0.36]	− 0.03 ± 0.07	[− 0.05, − 0.02]	< 0.01
	**LT**	0.21 ± 0.05	[0.20, 0.22]	0.21 ± 0.05	[0.20, 0.22]	− 0.01 ± 0.03	[− 0.01, 0.00]	0.07
	**cLF**	0.29 ± 0.06	[0.27, 0.30]	0.30 ± 0.07	[0.29, 0.32]	− 0.01 ± 0.06	[− 0.03, 0.00]	0.03

*SD*, standard deviation; *95% CI*, 95% confidence interval; *Diff*., mean difference; *HRC*, healthy reference cohort; *CL-JSN*, radiographically normal knees with joint space narrowing (JSN) in the contralateral (CL) knee; *CL-noROA*, radiographically normal knees without signs of radiographic OA (ROA) also in the contralateral knee, *DSC*, Dice similarity coefficient; *HD*, Hausdorff distance; *ASSD*, average symmetric surface distance; *VOE*, volume overlap error; *MT*, medial tibia; *cMF*, central medial femur; *LT*, lateral tibia; *cLF*, central lateral femur; *p*-values for differences between the All_E_ and the 1^st^_E_ U-Net computed using paired *t*-tests

**Fig. 4:**
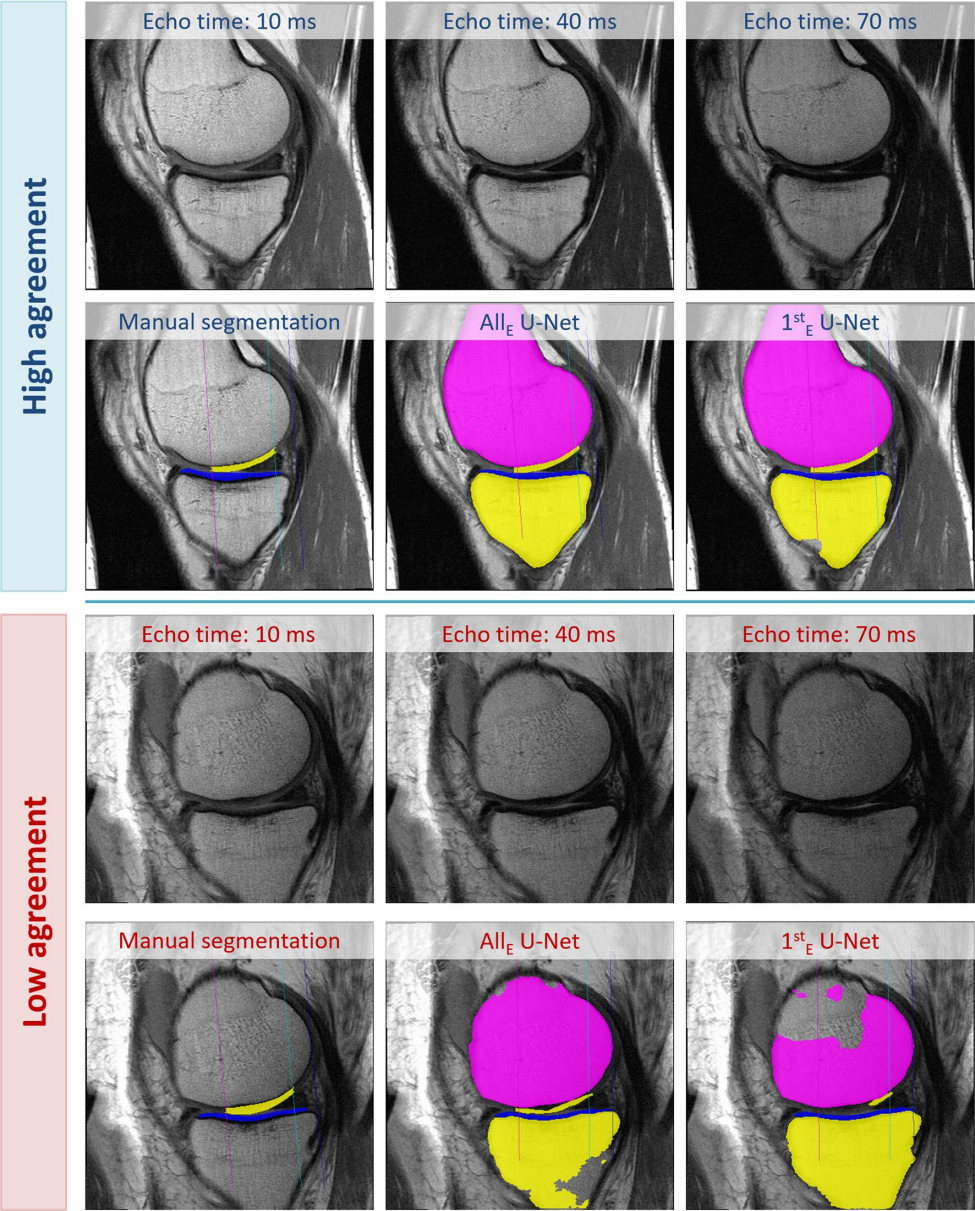
Illustration showing the first, middle, and last echo together with manual cartilage segmentations and U-Net-based bone and cartilage segmentations performed using both the U-Net trained from all (All_E_) and from the 1^st^_E_ echo (1^st^_E_). The example on the top shows a knee with high agreement of the cartilage segmentations, the example on the bottom shows a knee with low agreement of the cartilage segmentations

For cartilage segmentations, the agreement of the automated vs. the manual approaches (*n* = 88) was also high, and was similar for the All_E_ and the 1^st^_E_ U-Nets (Fig. 4). For both U-Nets, the DSC was greatest for the lateral tibia (0.88 ± 0.03 for both) and lowest for the central medial femur (All_E_/1^st^_E_: 0.82 ± 0.05/0.79 ± 0.06). A similar agreement was observed when analyzing the HRC test set, CL-JSN, and CL-noROA knees individually (Supplemental Table 2), and when using the re-trained U-Nets (Supplemental Table 3).

### Comparison of laminar T2 between U-Net and manual expert analysis

Laminar cartilage T2 from manual, All_E_, and 1^st^_E_ U-Net segmentations is reported in Table 2. Both the superficial and deep layer cartilage T2 from the All_E_ U-Net tended to be shorter than the superficial and deep layer T2 from manual expert segmentations, with most of the differences reaching statistical significance. The greatest T2 deviation was observed in both cartilage layers for the central medial femur (deep, − 2.2 ± 2.6 ms; superficial, − 1.1 ± 3.3 ms).

**Table 2:** Comparison of superficial and deep layer T2 times between manual cartilage segmentations and U-Net-based cartilage segmentations (all echoes (All_E_) U-Net & 1st echo (1^st^_E_) U-Net) in all 88 knees with manual segmentations (healthy reference cohort test set: *n* = 10, CL-JSN: *n* = 39, CL-noROA: *n* = 39)

	Manual	All_E_ U-Net					1^st^_E_ U-Net				
	Mean ± SD	Mean ± SD	Mean Diff	[95% CI]	*p*-value	*r*	Mean ± SD	Diff. ± SD	[95% CI]	*p*-value	*r*
Deep layer											
FTJ	37.2 ± 2.3	36.0 ± 1.9	− 1.2 ± 0.9	[− 1.4, − 1.0]	< 0.01	0.92	37.1 ± 2.4	− 0.1 ± 1.3	[− 0.4, 0.1]	0.34	0.85
MFTC	37.7 ± 2.7	36.5 ± 2.2	− 1.2 ± 1.3	[− 1.5, − 0.9]	< 0.01	0.88	37.8 ± 3.1	0.1 ± 2.3	[− 0.4, 0.6]	0.61	0.69
LFTC	36.7 ± 2.5	35.5 ± 2.1	− 1.1 ± 0.9	[− 1.3, − 0.9]	< 0.01	0.94	36.3 ± 2.4	− 0.4 ± 0.9	[− 0.6, − 0.2]	< 0.01	0.94
MT	33.5 ± 2.1	33.3 ± 2.0	− 0.3 ± 0.6	[− 0.4, − 0.1]	< 0.01	0.96	33.8 ± 2.0	0.3 ± 0.7	[0.1, 0.4]	< 0.01	0.95
cMF	41.9 ± 4.2	39.7 ± 3.2	− 2.2 ± 2.6	[− 2.7, − 1.6]	< 0.01	0.79	41.9 ± 5.8	0.0 ± 4.6	[− 1.0, 0.9]	0.94	0.62
LT	32.4 ± 2.2	31.7 ± 1.9	− 0.7 ± 0.8	[− 0.8, − 0.5]	< 0.01	0.94	32.3 ± 2.3	− 0.1 ± 0.9	[− 0.3, 0.1]	0.28	0.93
cLF	41.0 ± 3.7	39.4 ± 3.1	− 1.6 ± 1.4	[− 1.9, − 1.3]	< 0.01	0.93	40.3 ± 3.6	− 0.7 ± 1.4	[− 1.0, − 0.4]	< 0.01	0.92
Superficial layer											
FTJ	47.7 ± 3.6	47.1 ± 3.1	− 0.5 ± 1.7	[− 0.9, − 0.2]	< 0.01	0.89	49.2 ± 5.4	1.5 ± 3.2	[0.8, 2.2]	< 0.01	0.81
MFTC	48.1 ± 4.6	47.6 ± 3.5	− 0.5 ± 2.2	[− 1.0, 0.0]	< 0.03	0.89	50.4 ± 8.4	2.3 ± 5.6	[1.1, 3.5]	< 0.01	0.77
LFTC	47.2 ± 3.4	46.7 ± 3.3	− 0.6 ± 1.8	[− 0.9, − 0.2]	< 0.01	0.86	48.0 ± 4.5	0.8 ± 2.7	[0.2, 1.4]	0.01	0.79
MT	43.4 ± 3.3	43.5 ± 2.7	0.1 ± 1.6	[− 0.2, 0.4]	< 0.57	0.88	43.8 ± 3.8	0.4 ± 2.4	[− 0.1, 0.9]	0.12	0.78
cMF	52.8 ± 6.5	51.7 ± 4.8	− 1.1 ± 3.3	[− 1.8, − 0.4]	< 0.01	0.86	56.9 ± 14.1	4.1 ± 10.2	[2.0, 6.3]	< 0.01	0.74
LT	44.6 ± 3.3	44.0 ± 3.0	− 0.6 ± 1.9	[− 1.0, − 0.2]	< 0.01	0.83	44.6 ± 4.1	0.0 ± 2.5	[− 0.5, 0.6]	0.87	0.79
cLF	49.9 ± 4.2	49.4 ± 4.2	− 0.5 ± 2.2	[− 1.0, − 0.1]	< 0.03	0.86	51.5 ± 5.9	1.6 ± 3.7	[0.8, 2.3]	< 0.01	0.78

*CL-JSN*, radiographically normal knees with joint space narrowing (JSN) in the contralateral (CL) knee; *CL-noROA*, radiographically normal knees without signs of radiographic OA (ROA) also in the contralateral knee; *SD*, standard deviation; *95% CI*, 95% confidence interval; *r*, Pearson correlation coefficient; Diff., mean difference; *FTJ*, femorotibial joint; *MFTC*, medial femorotibial compartment; *LFTC*, lateral femorotibial compartment; *MT*, medial tibia; *cMF*, central medial femur; *LT*, lateral tibia; *cLF*, central lateral femur; *p*-values for differences between U-Net-based and manual cartilage T2 computed using paired *t*-tests

The superficial layer T2 tended to be longer for the 1^st^_E_ U-Net than for manual expert analysis, with several of the differences reaching statistical significance. The greatest difference was observed for the central medial femur (4.1 ± 10.2 ms), with this difference being mainly driven by the CL-JSN knees (Supplemental Table 4). Differences between deep layer T2 from the 1^st^_E_ U-Net and from manual expert analysis were less uniform and somewhat smaller than those observed for the All_E_ U-Net, with some of the differences reaching statistical significance. The greatest difference was observed for the central lateral femur (− 0.7 ± 1.4 ms).

Results for the HRC test set, CL-JSN, and CL-noROA knees individually are shown in Supplemental Table 4. The results for re-trained U-Nets were consistent with those observed in the main run for the All_E_ U-Net but showed larger deviations for the 1^st^E U-Net (Supplemental Table 5). Laminar T2 from both U-Nets was highly correlated with that from manual expert analysis (see Supplemental Fig. 2 for Bland & Altman plots). The lowest correlation was observed for the deep layer in the central medial femur for both U-Nets (All_E_: *r* = 0.79, 1^st^_E_: *r* = 0.62).

### Cross-sectional comparison between CL-JSN and CL-noROA knees

Results from the cross-sectional comparison between CL-JSN and CL-noROA knees are presented in Table 3 for the manual expert and the All_E_ U-Net analysis. Superficial layer T2 was longer in CL-JSN than in matched CL-noROA knees for FTJ, MFTC, and LFTC based on manual expert analysis, with the greatest Cohen’s *D* observed for the FTJ (0.48, 95% confidence interval (CI): [0.15, 0.81]), whereas deep layer differences did not reach statistical significance in the manual expert analysis (Cohen’s *D* FTJ: 0.28, 95% CI: [− 0.04, 0.60]). With the All_E_ U-Net, superficial and deep layer T2 were longer in CL-JSN knees than in the matched CL-noROA knees for FTJ, MFTC, and LFTC; Cohen’s *D* for the FTJ was 0.54 (95% CI: [0.20, 0.87]) in the superficial and 0.41 (95% CI: [0.08, 0.74]) in the deep layer. Results for all four femorotibial cartilages are shown in Supplemental Table 6.

**Table 3:** Comparison of superficial and deep layer T2 times at the year 1 visit (in ms) derived from manual and all echoes (All_E_) U-Net segmentations between matched CL-JSN (*n* = 39) and CL-noROA knees (*n* = 39) and between CL-JSN (*n* = 39) and non-matched CL-noROA knees (*n* = 982)

		CL-JSN	CL-noROA	CL-JSN vs. CL-noROA						
		Mean ± SD	Mean ± SD	Mean diff	[95% CI]	Cohen’s *D*	[95% CI]	*p*-value		
Matched comparison, manual segmentation										
Deep layer	**FTJ**	37.8 ± 2.3	37.1 ± 2.0	0.7	[− 0.1, 1.6]	0.28	[− 0.04, 0.60]	0.09		
	**MFTC**	38.4 ± 2.6	37.7 ± 2.4	0.6	[− 0.4, 1.7]	0.20	[− 0.12, 0.51]	0.23		
	**LFTC**	37.3 ± 2.6	36.5 ± 2.3	0.9	[− 0.2, 1.9]	0.27	[− 0.06, 0.58]	0.11		
Superficial layer	**FTJ**	49.0 ± 3.8	47.2 ± 2.8	1.8	[0.6, 3.0]	0.48	[0.15, 0.81]	< 0.01		
	**MFTC**	49.6 ± 4.7	47.6 ± 4.0	2.0	[0.4, 3.6]	0.40	[0.07, 0.72]	0.02		
	**LFTC**	48.4 ± 3.7	46.8 ± 2.7	1.5	[0.3, 2.8]	0.41	[0.08, 0.73]	0.01		
Matched comparison, All_E_ U-Net										
Deep layer	**FTJ**	36.7 ± 2.0	35.8 ± 1.7	0.9	[0.2, 1.6]	0.41	[0.08, 0.74]	0.01		
	**MFTC**	37.1 ± 2.3	36.3 ± 1.9	0.8	[0.0, 1.6]	0.31	[− 0.02, 0.63]	0.06		
	**LFTC**	36.2 ± 2.2	35.2 ± 1.9	1.0	[0.2, 1.8]	0.41	[0.08, 0.74]	0.01		
Superficial layer	**FTJ**	48.3 ± 3.2	46.5 ± 2.4	1.8	[0.7, 2.9]	0.54	[0.20, 0.87]	< 0.01		
	**MFTC**	48.9 ± 3.6	46.9 ± 2.7	2.0	[0.8, 3.2]	0.54	[0.20, 0.87]	< 0.01		
	**LFTC**	47.7 ± 3.4	46.1 ± 2.9	1.6	[0.3, 2.9]	0.40	[0.07, 0.72]	0.02		
Non-matched comparison, All_E_ U-Net										
Deep layer	**FTJ**	36.7 ± 2.0	36.1 ± 1.9	0.6	[0.0, 1.2]	0.30	[− 0.02, 0.63]	0.06		
	**MFTC**	37.1 ± 2.3	36.6 ± 2.2	0.5	[− 0.2, 1.2]	0.24	[− 0.08, 0.56]	0.14		
	**LFTC**	36.2 ± 2.2	35.6 ± 2.0	0.6	[0.0, 1.3]	0.31	[− 0.01, 0.63]	0.06		
Superficial layer	**FTJ**	48.3 ± 3.2	46.9 ± 2.7	1.5	[0.6, 2.3]	0.54	[0.22, 0.86]	< 0.01		
	**MFTC**	48.9 ± 3.6	47.3 ± 3.2	1.6	[0.5, 2.6]	0.49	[0.17, 0.81]	< 0.01		
	**LFTC**	47.7 ± 3.4	46.4 ± 2.8	1.4	[0.5, 2.3]	0.49	[0.16, 0.81]	< 0.01		

*CL-JSN*, radiographically normal knees with joint space narrowing (JSN) in the contralateral (CL) knee; *CL-noROA*, radiographically normal knees without signs of radiographic OA (ROA) also in the contralateral knee; *SD*, standard deviation; *95% CI*, 95% confidence interval; *r*, Pearson correlation coefficient; *FTJ*, femorotibial joint; *MFTC*, medial femorotibial compartment; *LFTC*, lateral femorotibial compartment; *MT*, medial tibia; *cMF*, central medial femur; *LT*, lateral tibia; *cLF*, central lateral femur; *Mean diff*., mean difference; *p*-values for differences between CL-JSN and CL-noROA knees computed using paired (matched comparisons) or unpaired *t*-tests (non-matched comparison)

The results observed in the non-matched comparison using the All_E_ U-Net were similar to those from the matched comparison. The effect size for differences between CL-JSN and CL-noROA knees in the FTJ was 0.54 (95% CI: [0.22, 0.86]) in the superficial and 0.30 (95% CI: [− 0.02, 0.63]) in the deep layer.

### Longitudinal comparison between CL-JSN and CL-noROA knees

Results from the longitudinal comparison between CL-JSN and CL-noROA knees are presented in Table 4 for the manual expert and the All_E_ U-Net analysis. Differences in 3-year change of superficial layer T2 between CL-JSN and matched CL-noROA knees were only minute (Cohen’s *D* ≤ 0.10) and not statistically significant for manual expert or U-Net analyses for any knee region. Both analysis methods showed a greater increase in deep layer T2 in the FTJ and LFTC (but not the MFTC) over 3 years in the CL-JSN knees compared with the matched CL-noROA knees. Cohen’s *D* for deep-layer FTJ differences between CL-JSN and matched CL-noROA knees was 0.37 (95% CI: [0.04, 0.69]) for manual expert analysis and 0.47 (95% CI: [0.14, 0.80]) for the All_E_ U-Net. Results for the four femorotibial cartilages are shown in Supplemental Table 7.

**Table 4:** Comparison of superficial and deep layer T2 change between the year 1 and the year 4 visits (in ms) derived from manual and all echoes (All_E_) U-Net segmentations between matched CL-JSN (*n* = 39) and CL-noROA knees (*n* = 39) and between CL-JSN (*n* = 39) and non-matched CL-noROA knees (*n* = 777)

		CL-JSN	CL-noROA		CL-JSN vs. CL-noROA					
		Mean ± SD	Mean ± SD		Mean diff	[95% CI]	Cohen’s *D*	[95% CI]		*p*-value
Matched comparison, manual segmentation										
Deep layer	**FTJ**	1.2 ± 2.5	0.1 ± 1.8		1.1	[0.1, 2.0]	0.37	[0.04, 0.69]		0.03
	**MFTC**	1.4 ± 3.4	0.4 ± 2.4		1.0	[− 0.4, 2.4]	0.24	[− 0.08, 0.55]		0.15
	**LFTC**	1.0 ± 2.5	− 0.1 ± 1.8		1.2	[0.2, 2.1]	0.41	[0.08, 0.73]		0.01
Superficial layer	**FTJ**	0.9 ± 1.9	0.9 ± 1.5		0.0	[− 0.8, 0.8]	0.01	[− 0.31, 0.32]		0.97
	**MFTC**	1.3 ± 2.5	1.3 ± 2.2		0.0	[− 1.0, 1.0]	0.01	[− 0.31, 0.32]		0.97
	**LFTC**	0.5 ± 2.2	0.5 ± 1.9		0.0	[− 1.1, 1.1]	0.00	[− 0.31, 0.32]		0.98
Matched comparison, All_E_ U-Net										
Deep layer	**FTJ**	0.8 ± 1.7	− 0.3 ± 1.5		1.1	[0.3, 1.8]	0.47	[0.14, 0.80]		0.01
	**MFTC**	0.8 ± 1.8	0.0 ± 1.9		0.8	[0.0, 1.7]	0.32	[− 0.01, 0.64]		0.05
	**LFTC**	0.8 ± 2.1	− 0.5 ± 1.6		1.3	[0.5, 2.2]	0.51	[0.17, 0.84]		< 0.01
Superficial layer	**FTJ**	0.5 ± 1.5	0.5 ± 1.3		0.0	[− 0.6, 0.7]	0.02	[− 0.29, 0.33]		0.90
	**MFTC**	0.7 ± 1.7	0.9 ± 1.9		− 0.2	[− 1.0, 0.6]	− 0.07	[− 0.38, 0.24]		0.66
	**LFTC**	0.3 ± 1.9	0.1 ± 1.5		0.3	[− 0.6, 1.1]	0.10	[− 0.22, 0.41]		0.55
Non-matched comparison, All_E_ U-Net										
Deep layer	**FTJ**	0.8 ± 1.7	0.3 ± 1.7		0.5	[0.0, 1.1]	0.31	[− 0.01, 0.63]		0.06
	**MFTC**	0.8 ± 1.8	0.4 ± 2.0		0.4	[− 0.2, 1.0]	0.20	[− 0.13, 0.52]		0.23
	**LFTC**	0.8 ± 2.1	0.1 ± 1.8		0.7	[0.1, 1.3]	0.39	[0.07, 0.72]		0.02
Superficial layer	**FTJ**	0.5 ± 1.5	0.5 ± 1.4		0.0	[− 0.5, 0.4]	− 0.01	[− 0.33, 0.32]		0.97
	**MFTC**	0.7 ± 1.7	0.7 ± 1.8		0.0	[− 0.6, 0.6]	− 0.01	[− 0.33, 0.31]		0.95
	**LFTC**	0.3 ± 1.9	0.3 ± 1.4		0.0	[− 0.5, 0.5]	0.00	[− 0.32, 0.32]		1.00

*CL-JSN*, radiographically normal knees with joint space narrowing (JSN) in the contralateral (CL) knee; *CL-noROA*, radiographically normal knees without signs of radiographic OA (ROA) also in the contralateral knee; *SD*, standard deviation; *95% CI*, 95% confidence interval; *r*, Pearson correlation coefficient; *FTJ*, femorotibial joint; *MFTC*, medial femorotibial compartment; *LFTC*, lateral femorotibial compartment; *MT*, medial tibia; *cMF*, central medial femur; *LT*, lateral tibia; *cLF*, central lateral femur; *Diff*., mean difference; *p*-values for differences between CL-JSN and CL-noROA knees computed using paired (matched comparisons) or unpaired *t*-tests (non-matched comparison)

In the non-matched comparison, no statistically significant differences were observed for 3-year change in superficial T2 between CL-JSN and CL-noROA knees in any region using the All_E_ U-Net. CL-JSN knees showed a greater deep layer T2 increase than CL-noROA knees in the LFTC. The effect size for FTJ was close to 0 in the superficial layer and was 0.31 (95% CI: [− 0.01, 0.63]) in the deep layer.

## Discussion

This study aimed to technically and clinically validate a fully automated laminar cartilage T2 analysis methodology using a U-Net CNN. The results showed a high agreement between U-Net and manual expert segmentations, along with satisfactory accuracy in U-Net-based laminar T2 analysis across both deep and superficial cartilage layers. Importantly, the U-Net-based T2 analysis exhibited similar sensitivity to cross-sectional and longitudinal differences between CL-JSN and CL-noROA knees in an early OA model as previously reported for a manual expert analysis [10]. These results suggest that the U-Net CNN approach is well-suited for fully automated laminar T2 analyses in very large cohorts.

The agreement between automated and manual expert segmentations observed in this study was comparable to that previously reported by other DL studies for cartilage morphometry using high resolution 3D MRIs (DSC between ~ 0.80 and 0.90) [11, 12]. Yet, even somewhat higher DSCs were observed by us when using a similar U-Net approach as in the current study for automated cartilage thickness analyses from 3D MRIs (DSCs 0.89 to 0.92) [13]. The current findings are also at the upper end of the range previously reported for MESE MRIs in DL-based studies (DSC 0.68 to 0.85) [15, 17]. Interestingly, the segmentation agreement in this study was consistently lower for femoral than for tibial cartilage, mirroring observations from U-Net-based cartilage segmentations from high resolution 3D MRI [13]. This somewhat lower agreement may be due to the greater curvature of the femoral surface, which leads to greater partial volume effects, as opposed to the relatively flat tibial plateau. Additionally, the smaller size of the central femoral cartilage region (about half the number of the tibial voxels) results in a larger relative impact on agreement metrics when segmentation inaccuracies occur [24].

**Fig. 5:**
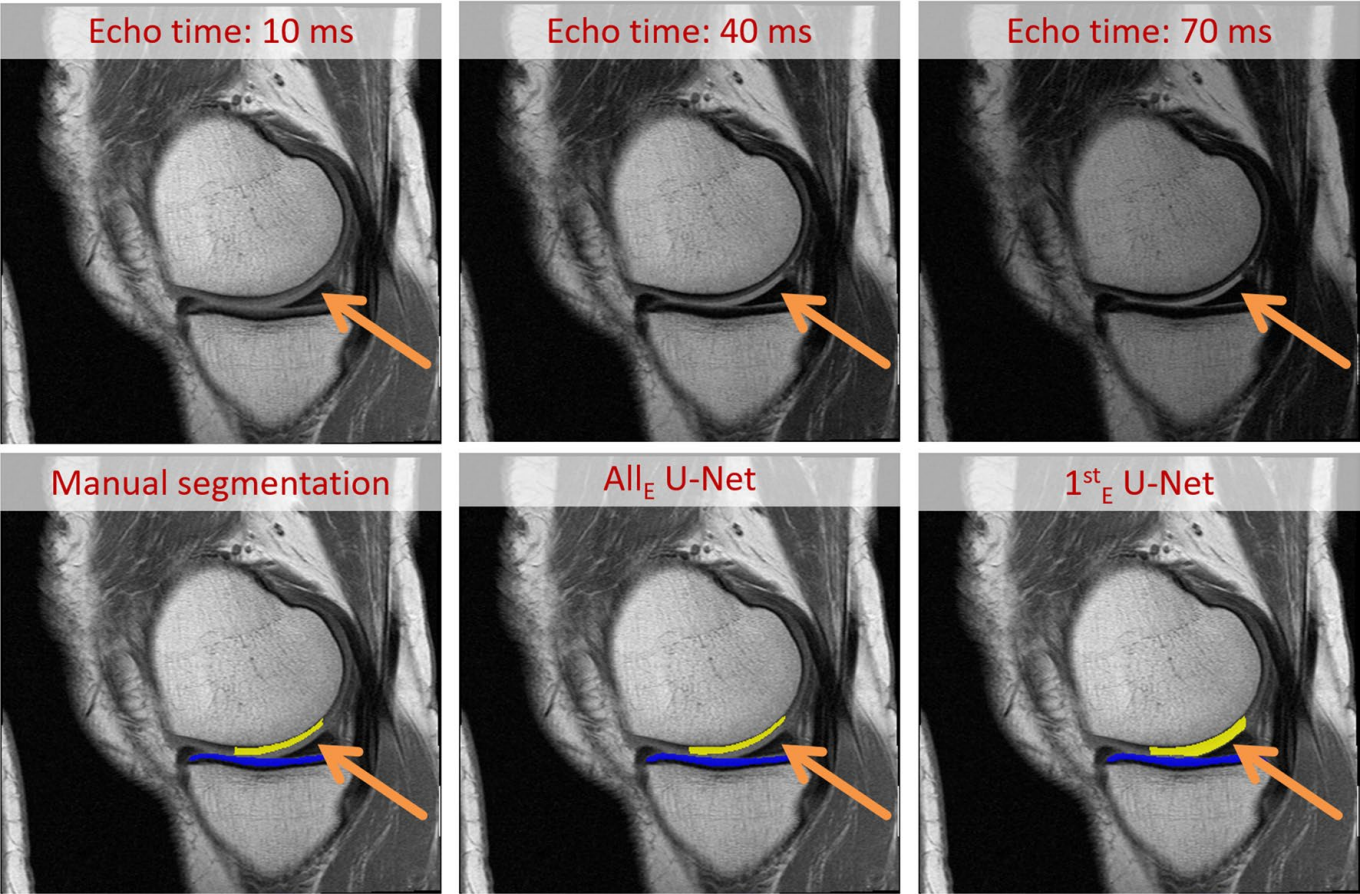
Illustration of an incorrect segmentation of the central medial femur performed by the 1^st^_E_ U-Net in knees with accumulations of synovial fluid, which can be better distinguished from cartilage in later echoes (e.g., 40 ms or higher). The arrows indicate the location of the incorrect segmentation

The 1^st^_E_ U-Net showed somewhat lower accuracy than the All_E_ U-Net, particularly in the central medial femur of CL-JSN knees. This may be due to the lower contrast between cartilage and synovial fluid in the 1st echo, resulting in slight over-segmentation in the presence of synovial fluid (Fig. 5). Since contrast between articular cartilage and synovial fluid increases with greater echo time, future automated laminar T2 analyses should incorporate the information from all seven MESE MRI echoes as also done in manual expert analyses.

The accuracy of the T2 analysis observed in the current study for the All_E_ U-Net was within the range as that reported for MESE MRI by Thomas et al.[15] (up to − 2.6 ms) and Liu et al. [16] (up to 1.1 ms), but higher than that reported by Razmjoo et al. (up to 0.20 ms) [17]. The greatest deviation between automated and manual T2 with the All_E_ U-Net was observed for the deep layer of the central medial femur (− 2.2 ms), which is the same region and layer that also showed the greatest difference in the study by Thomas et al. (− 2.6 ms) [15].

Previous studies using manual cartilage segmentations observed that superficial layer T2 is longer and longitudinal increase in deep layer T2 is greater in CL-JSN knees than in matched CL-noROA knees in this early OA model [10]. The U-Net-based laminar T2 analysis showed comparable sensitivity to cross-sectional and longitudinal differences between CL-JSN and matched CL-noROA knees. Additionally, it was also sensitive to cross-sectional differences in deep layer T2, which did not reach statistical significance and the same effect size with manual expert analyses. The effect sizes observed for the All_E_ U-Net tended to be greater than that observed for the manual analyses, potentially due of the lower T2 variability between participants.

All 1847 MESE MRIs could be automatically analyzed using the cartilage T2 analysis methodology, without any manual correction required. This is of great promise for the future use of this methodology in very large cohorts. As expected, effect sizes in the non-matched comparison were comparable to those observed in the matched comparison, as effect sizes are not sample size dependent. Fully automated T2 analyses may therefore allow to gain high statistical power in future trials encompassing large samples, without the need for manual expert segmentation, quality control readings, and potential corrections of the segmentations, a process that takes considerable time and human as well as financial resources.

One limitation of this study is the considerable number of missing follow-up visits in the longitudinal analysis. However, about 80% of the CL-noROA knees had complete longitudinal data available, providing a more than sufficient sample size to investigate the effect size for longitudinal cartilage T2 differences between CL-JSN and CL-noROA knees. Another limitation of the study is that, in accordance with the study design, only knees without radiographic OA were analyzed, so the performance of the U-Net-based T2 analysis in knees with radiographic signs of OA is yet unknown. However, about 45% of the knees included in the current study displayed cartilage damage according to semi-quantitative readings performed by expert radiologists and thus showed early OA changes [25]. Also, cartilage T2 analysis is generally applied to knees without or with not more than early (radiographic) OA, because the loss of superficial cartilage tissue with the longest T2 relaxation times impairs the ability to detect prolongation of T2 in knees with advanced, progressive OA [26].

In conclusion, the U-Net CNN-based segmentation of femorotibial cartilages from MESE MRI exhibited a high level of segmentation agreement compared to manual expert analysis. Furthermore, the accuracy in measuring laminar T2 was comparable to that reported in previous studies. The All_E_ U-Net seems particularly suitable for automated analyses, as it leverages information from all available MESE MRI echoes. The technique used for this study was found to be sensitive to laminar cartilage T2 differences in an early OA model comparing radiographically normal knees with vs. without contralateral JSN (or other signs of radiographic OA) both cross-sectionally and longitudinally. Moreover, it successfully analyzed a total of 1847 MESE MRIs, demonstrating its robustness for potential application to data from large cohorts for future studies and clinical trials.

## Supplementary Information

Below is the link to the electronic supplementary material.

Supplementary file1 (DOCX 1918 KB)

## Data Availability

The data that support the findings of this study are available for scientific purposes from the corresponding author upon reasonable request.
