# Supplementary material for: Evaluation of an automated laminar cartilage T2 relaxation time analysis method in an early osteoarthritis model
Source: Skeletal Radiol. 2024 Sep 4;54(3):571–84. doi: 10.1007/s00256-024-04786-1 (PMC11769870; doi:10.1007/s00256-024-04786-1)

## Supplement 1: Automated computation of the femoral Region of Interest (ROI)

Methodology

The position of the fibula allows to distinguish between the cartilages in the medial (MFTC) and the lateral compartment (LFTC) was determined through automated fibular bone segmentations. After detecting the fibula, an automated software tool was used to separate the segmented tibial and femoral cartilages into the medial and lateral tibial cartilage and into the medial and lateral femoral cartilage.

The femoral ROI used for separating the central (weight-bearing) part of the femoral condyles from the femoral trochlea and from the posterior parts of the femoral condyles is constructed from a line parallel to the femoral shaft through the trochlear notch and from a line connecting the most posterior points of the medial and the lateral femoral condyles. These two lines allow computing the plane through the trochlear notch (anterior separation) as well as the parallel plane at 75% of the distance between the trochlear notch and the most posterior points of both femoral condyles.

The algorithmic detection of the femoral ROI relied on the automated bone segmentations in the sagittal MRIs and reproduced the steps performed by human readers for setting the femoral ROI. The most posterior points of the medial and lateral femoral condyle were identified by determining the first, the last, and the middle slice containing segmentations labeled as femoral bone, and by then identifying the two most posterior femoral bone points in the range between the first and the middle slice, and between the middle and the last slice containing femoral bone segmentations. The position of the fibula was then used for labeling these points as posterior endpoints of either the medial or lateral femoral condyle. The anterior border of the femoral ROI is defined by the trochlear notch of the femur and was identified by first determining the slice between the femoral condyles, in which the most distal row comprising femoral bone segmentation has the least distal extent. In this slice, the 2 adjacent slices towards the medial femoral condyle, and the 2 slices towards the lateral femoral condyle, the most posterior point was identified at the height of the least distal extent of the femoral bone segmentation, minus an offset of 2.4mm in each of the 5 slices. This offset has been shown to provide the best replication of the manually set trochlear notch position. From these 5 points, the most anterior one was chosen to determine the position of the trochlear notch. The anterior ROI line was then defined as the line through this position of the trochlear notch, and through the point located at 70% of the anterior to posterior distance of the bone shaft, at a height 30 pixel below the most superior segmentation of the femoral bone. If no suitable bone shaft segmentation was available (e.g., because motion artefacts precluded a complete bone segmentation), a vertical line through the trochlear notch position was used as anterior ROI line.

Agreement between computed and manually set femoral region of interest

The position of the femoral region of interest was successfully detected in all knees. The automatically computed position of the posterior endpoint of the medial femoral condyle deviated from the manually set position by 1.6±1.1mm in the HRC test set and by 1.9±1.4mm in the radiographically normal knees with joint space narrowing (JSN) in the contralateral (CL) knee (CL-JSN) and the matched radiographically normal knees without signs of radiographic OA (ROA) also in the contralateral knee knees (CL-noROA).

**Table: Comparison of computed femoral region of interest (ROI) vs. manually set femoral ROI (in mm)**

|  | | **Healthy reference  test set (n=10)** | | | **Matched CL-JSN &  CL-noROA knees (n=78)** | | |
| --- | --- | --- | --- | --- | --- | --- | --- |
|  |  | **Mean ± SD** | **Min** | **Max** | **Mean ± SD** | **Min** | **Max** |
| **Posterior end of medial femur** | **Difference X** | -0.6 ± 0.2 | -0.9 | -0.3 | -0.4 ± 0.3 | -1.3 | 0.6 |
|  | **Difference Y** | -1.2 ± 1.2 | -4.1 | 0.3 | -0.9 ± 2.0 | -6.9 | 4.1 |
|  | **Difference Z** | -1.1 ± 1.7 | -3.5 | 0.0 | -1.2 ± 2.4 | -7.0 | 7.0 |
|  | **Abs. diff.** | 1.6 ± 1.1 | 0.5 | 4.5 | 1.9 ± 1.4 | 0.0 | 7.5 |
| **Posterior end of lateral femur** | **Difference X** | -0.6 ± 0.2 | -0.9 | -0.3 | -0.3 ± 0.4 | -1.3 | 1.6 |
|  | **Difference Y** | -1.5 ± 1.7 | -4.7 | 0.6 | -0.2 ± 1.9 | -5.3 | 5.0 |
|  | **Difference Z** | -3.5 ± 2.9 | -7.0 | 0.0 | -3.0 ± 3.2 | -10.5 | 3.5 |
|  | **Abs. diff.** | 2.1 ± 1.5 | 0.5 | 5.1 | 1.9 ± 1.2 | 0.0 | 5.8 |
| **Anterior ROI line** | **Difference X** | -0.9 ± 1.0 | -3.0 | 0.1 | 0.4 ± 1.2 | -3.5 | 3.0 |
|  | **Abs. diff.** | 1.0 ± 1.0 | 0.1 | 3.0 | 1.0 ± 0.8 | 0.0 | 3.5 |

SD: standard deviation, Min: minimum difference, Max.: maximum difference, Abs. diff.: Absolute difference

Similar differences were observed for the posterior endpoint of the lateral femoral condyle in the HRC test set (2.1±1.5mm) and the matched CL-JSN and CL-noROA knees (1.9±1.2mm). The differences for the anterior line were evaluated at the height of the femoral cartilage segmentations in the slice, in which the trochlea of the femur was detected, and amounted to 1.0±1.0mm for the HRC test set and to 1.0±0.08mm for the matched CL-JSN and CL-noROA knees.

**Supplemental Table 1: Demographic data**

|  | | **Healthy reference cohort test set (n=10)** | | **CL-JSN knees (n=39)** | | **Matched CL-noROA knees (n=39)** | | **All CL-noROA knees (n=982)** | |
| --- | --- | --- | --- | --- | --- | --- | --- | --- | --- |
|  |  | **Mean/N** | **SD/%** | **Mean/N** | **SD/%** | **Mean/N** | **SD/%** | **Mean/N** | **SD/%** |
| **Age** | (years) | 52.0 | 9.5 | 67.1 | 8.5 | 67.2 | 8.3 | 59.8 | 8.9 |
| **BMI** | (kg/m²) | 24.3 | 4.0 | 28.1 | 3.9 | 27.9 | 3.6 | 27.0 | 4.5 |
| **Sex** | Women (N/%) | 6 | 60.0 | 16 | 41.0 | 16 | 41.0 | 571 | 58.1 |
|  | Men (N/%) | 4 | 40.0 | 23 | 59.0 | 23 | 59.0 | 411 | 41.9 |
| **KLG right** | 0 (N/%) | 10 | 100.0 | 39 | 100.0 | 39 | 100.0 | 982 | 100.0 |
| **KLG left** | 0 (N/%) | 10 | 100.0 | 0 | 0.0 | 39 | 100.0 | 982 | 100.0 |
|  | 2 (N/%) | 0 | 0.0 | 22 | 56.4 | 0 | 0.0 | 0 | 0.0 |
|  | 3 (N/%) | 0 | 0.0 | 13 | 33.3 | 0 | 0.0 | 0 | 0.0 |
|  | 4 (N/%) | 0 | 0.0 | 4 | 10.3 | 0 | 0.0 | 0 | 0.0 |
| **Med. JSN right** | 0 (N/%) | 10 | 100.0 | 39 | 100.0 | 39 | 100.0 | 982 | 100.0 |
| **Lat. JSN right** | 0 (N/%) | 10 | 100.0 | 39 | 100.0 | 39 | 100.0 | 982 | 100.0 |
| **Med. JSN left** | 0 (N/%) | 0 | 0.0 | 7 | 17.9 | 39 | 100.0 | 982 | 100.0 |
|  | 1 (N/%) | 0 | 0.0 | 17 | 43.6 | 0 | 0.0 | 0 | 0.0 |
|  | 2 (N/%) | 0 | 0.0 | 12 | 30.8 | 0 | 0.0 | 0 | 0.0 |
|  | 3 (N/%) | 0 | 0.0 | 3 | 7.7 | 0 | 0.0 | 0 | 0.0 |
| **Lat. JSN left** | 0 (N/%) | 10 | 100.0 | 32 | 82.1 | 39 | 100.0 | 982 | 100.0 |
|  | 1 (N/%) | 0 | 0.0 | 5 | 12.8 | 0 | 0.0 | 0 | 0.0 |
|  | 2 (N/%) | 0 | 0.0 | 1 | 2.6 | 0 | 0.0 | 0 | 0.0 |
|  | 3 (N/%) | 0 | 0.0 | 1 | 2.6 | 0 | 0.0 | 0 | 0.0 |

CL-JSN: radiographically normal knees with joint space narrowing (JSN) in the contralateral (CL) knee, CL-noROA: radiographically normal knees without signs of radiographic OA (ROA) also in the contralateral knee, SD: standard deviation, KLG: Kellgren & Lawrence grade (scale: 0…4), Med./lat. JSN: Medial/lateral OARSI joint space narrowing grade (scale: 0…3)

**Supplemental Table 3: Agreement between U-Net-based and manual cartilage segmentations from multi-echo spin-echo MRIs and comparison of the agreement between the all-echoes (All_E_) U-Net and the first-echo (1^st^_E_) U-Net in 10 knees from the healthy reference cohort (HRC) test set, 39 CL-JSN, and 39 matched CL-noROA knees**

|  |  | | **All_E_ U-Net** | | **First echo U-Net** | | **All_E_ vs. 1^st^ echo U-Net** | | |
| --- | --- | --- | --- | --- | --- | --- | --- | --- | --- |
|  |  |  | **Mean ± SD** | **95% CI** | **Mean ± SD** | **95% CI** | **Diff. ± SD** | **95% CI** | **p-value** |
| **HRC test set (n=10)** | **DSC** | **MT** | 0.86 ± 0.03 | [0.84, 0.88] | 0.85 ± 0.03 | [0.83, 0.87] | 0.01 ± 0.01 | [0.00, 0.01] | 0.11 |
|  |  | **cMF** | 0.82 ± 0.05 | [0.79, 0.86] | 0.81 ± 0.06 | [0.77, 0.85] | 0.01 ± 0.02 | [0.00, 0.03] | 0.05 |
|  |  | **LT** | 0.89 ± 0.02 | [0.87, 0.90] | 0.88 ± 0.03 | [0.86, 0.90] | 0.01 ± 0.02 | [0.00, 0.02] | 0.23 |
|  |  | **cLF** | 0.84 ± 0.02 | [0.83, 0.86] | 0.84 ± 0.03 | [0.82, 0.86] | 0.00 ± 0.03 | [-0.02, 0.02] | 0.82 |
|  | **HD (mm)** | **MT** | 3.63 ± 1.13 | [2.83, 4.44] | 4.45 ± 1.52 | [3.36, 5.53] | -0.81 ± 1.40 | [-1.81, 0.19] | 0.10 |
|  |  | **cMF** | 4.72 ± 1.80 | [3.44, 6.01] | 4.39 ± 1.90 | [3.03, 5.74] | 0.34 ± 1.48 | [-0.72, 1.40] | 0.49 |
|  |  | **LT** | 4.13 ± 1.05 | [3.38, 4.88] | 3.99 ± 1.15 | [3.17, 4.81] | 0.14 ± 1.46 | [-0.90, 1.19] | 0.76 |
|  |  | **cLF** | 4.50 ± 1.71 | [3.28, 5.72] | 3.63 ± 1.37 | [2.65, 4.61] | 0.87 ± 1.89 | [-0.48, 2.23] | 0.18 |
|  | **ASSD (mm)** | **MT** | 0.24 ± 0.16 | [0.12, 0.35] | 0.26 ± 0.15 | [0.16, 0.37] | -0.03 ± 0.11 | [-0.10, 0.05] | 0.47 |
|  |  | **cMF** | 0.35 ± 0.23 | [0.19, 0.51] | 0.37 ± 0.24 | [0.20, 0.54] | -0.02 ± 0.09 | [-0.08, 0.04] | 0.42 |
|  |  | **LT** | 0.22 ± 0.10 | [0.15, 0.29] | 0.20 ± 0.10 | [0.13, 0.28] | 0.01 ± 0.05 | [-0.02, 0.05] | 0.44 |
|  |  | **cLF** | 0.19 ± 0.08 | [0.13, 0.25] | 0.19 ± 0.09 | [0.13, 0.26] | 0.00 ± 0.12 | [-0.09, 0.08] | 0.92 |
|  | **VOE**  **(%)** | **MT** | 0.25 ± 0.05 | [0.22, 0.28] | 0.26 ± 0.05 | [0.22, 0.29] | -0.01 ± 0.02 | [-0.02, 0.00] | 0.13 |
|  |  | **cMF** | 0.30 ± 0.07 | [0.25, 0.35] | 0.32 ± 0.08 | [0.26, 0.38] | -0.02 ± 0.03 | [-0.04, 0.00] | 0.06 |
|  |  | **LT** | 0.20 ± 0.04 | [0.17, 0.23] | 0.21 ± 0.05 | [0.18, 0.25] | -0.01 ± 0.02 | [-0.03, 0.01] | 0.22 |
|  |  | **cLF** | 0.27 ± 0.03 | [0.25, 0.29] | 0.27 ± 0.04 | [0.24, 0.30] | 0.00 ± 0.04 | [-0.03, 0.03] | 0.85 |
| **CL-JSN knees (n=39)** | **DSC** | **MT** | 0.87 ± 0.03 | [0.87, 0.88] | 0.86 ± 0.03 | [0.86, 0.87] | 0.01 ± 0.02 | [0.01, 0.02] | 0.00 |
|  |  | **cMF** | 0.82 ± 0.04 | [0.80, 0.83] | 0.79 ± 0.06 | [0.77, 0.81] | 0.03 ± 0.06 | [0.01, 0.04] | 0.01 |
|  |  | **LT** | 0.88 ± 0.03 | [0.87, 0.89] | 0.88 ± 0.03 | [0.87, 0.89] | 0.00 ± 0.02 | [0.00, 0.01] | 0.68 |
|  |  | **cLF** | 0.84 ± 0.04 | [0.82, 0.85] | 0.83 ± 0.05 | [0.81, 0.85] | 0.01 ± 0.05 | [-0.01, 0.02] | 0.31 |
|  | **HD (mm)** | **MT** | 4.39 ± 1.82 | [3.80, 4.97] | 4.23 ± 1.03 | [3.89, 4.56] | 0.16 ± 1.49 | [-0.33, 0.64] | 0.51 |
|  |  | **cMF** | 4.67 ± 1.87 | [4.07, 5.28] | 4.52 ± 1.73 | [3.96, 5.08] | 0.15 ± 1.68 | [-0.40, 0.69] | 0.59 |
|  |  | **LT** | 4.25 ± 1.17 | [3.87, 4.63] | 3.82 ± 0.87 | [3.54, 4.11] | 0.42 ± 1.37 | [-0.02, 0.87] | 0.06 |
|  |  | **cLF** | 4.29 ± 1.79 | [3.71, 4.87] | 4.56 ± 1.92 | [3.93, 5.18] | -0.27 ± 2.32 | [-1.02, 0.48] | 0.47 |
|  | **ASSD (mm)** | **MT** | 0.17 ± 0.12 | [0.14, 0.21] | 0.21 ± 0.10 | [0.17, 0.24] | -0.03 ± 0.08 | [-0.06, -0.01] | 0.02 |
|  |  | **cMF** | 0.36 ± 0.27 | [0.27, 0.45] | 0.42 ± 0.27 | [0.33, 0.51] | -0.06 ± 0.23 | [-0.13, 0.02] | 0.12 |
|  |  | **LT** | 0.19 ± 0.10 | [0.16, 0.23] | 0.18 ± 0.09 | [0.15, 0.21] | 0.01 ± 0.09 | [-0.02, 0.04] | 0.34 |
|  |  | **cLF** | 0.23 ± 0.18 | [0.17, 0.29] | 0.26 ± 0.24 | [0.18, 0.34] | -0.03 ± 0.29 | [-0.13, 0.06] | 0.47 |
|  | **VOE**  **(%)** | **MT** | 0.22 ± 0.04 | [0.21, 0.24] | 0.24 ± 0.04 | [0.23, 0.25] | -0.02 ± 0.03 | [-0.03, -0.01] | 0.00 |
|  |  | **cMF** | 0.31 ± 0.06 | [0.29, 0.33] | 0.34 ± 0.08 | [0.32, 0.37] | -0.03 ± 0.07 | [-0.06, -0.01] | 0.01 |
|  |  | **LT** | 0.21 ± 0.05 | [0.19, 0.22] | 0.21 ± 0.05 | [0.19, 0.23] | 0.00 ± 0.03 | [-0.01, 0.01] | 0.67 |
|  |  | **cLF** | 0.28 ± 0.06 | [0.26, 0.30] | 0.29 ± 0.07 | [0.27, 0.31] | -0.01 ± 0.07 | [-0.03, 0.01] | 0.32 |
| **CL-noROA knees (n=39)** | **DSC** | **MT** | 0.88 ± 0.03 | [0.87, 0.88] | 0.86 ± 0.03 | [0.85, 0.87] | 0.01 ± 0.02 | [0.01, 0.02] | 0.00 |
|  |  | **cMF** | 0.81 ± 0.05 | [0.80, 0.83] | 0.79 ± 0.07 | [0.77, 0.81] | 0.03 ± 0.05 | [0.01, 0.04] | 0.00 |
|  |  | **LT** | 0.89 ± 0.03 | [0.88, 0.89] | 0.88 ± 0.03 | [0.87, 0.89] | 0.01 ± 0.02 | [0.00, 0.01] | 0.12 |
|  |  | **cLF** | 0.82 ± 0.05 | [0.81, 0.84] | 0.81 ± 0.05 | [0.79, 0.82] | 0.01 ± 0.04 | [0.00, 0.03] | 0.02 |
|  | **HD (mm)** | **MT** | 3.97 ± 1.12 | [3.61, 4.33] | 3.93 ± 1.04 | [3.60, 4.27] | 0.04 ± 1.00 | [-0.29, 0.36] | 0.81 |
|  |  | **cMF** | 4.63 ± 1.78 | [4.05, 5.20] | 4.72 ± 1.58 | [4.20, 5.23] | -0.09 ± 1.29 | [-0.51, 0.33] | 0.67 |
|  |  | **LT** | 4.80 ± 5.41 | [3.05, 6.55] | 4.69 ± 5.49 | [2.92, 6.47] | 0.11 ± 1.35 | [-0.33, 0.54] | 0.63 |
|  |  | **cLF** | 4.35 ± 1.50 | [3.86, 4.84] | 4.46 ± 1.12 | [4.10, 4.82] | -0.11 ± 1.63 | [-0.64, 0.42] | 0.67 |
|  | **ASSD (mm)** | **MT** | 0.16 ± 0.12 | [0.13, 0.20] | 0.18 ± 0.14 | [0.14, 0.23] | -0.02 ± 0.09 | [-0.05, 0.01] | 0.16 |
|  |  | **cMF** | 0.30 ± 0.22 | [0.23, 0.37] | 0.39 ± 0.25 | [0.31, 0.47] | -0.09 ± 0.20 | [-0.16, -0.03] | 0.00 |
|  |  | **LT** | 0.18 ± 0.08 | [0.16, 0.21] | 0.19 ± 0.12 | [0.15, 0.22] | 0.00 ± 0.10 | [-0.03, 0.03] | 0.91 |
|  |  | **cLF** | 0.24 ± 0.18 | [0.18, 0.30] | 0.26 ± 0.15 | [0.22, 0.31] | -0.02 ± 0.16 | [-0.08, 0.03] | 0.38 |
|  | **VOE**  **(%)** | **MT** | 0.22 ± 0.04 | [0.21, 0.23] | 0.24 ± 0.04 | [0.23, 0.26] | -0.02 ± 0.03 | [-0.03, -0.01] | 0.00 |
|  |  | **cMF** | 0.31 ± 0.07 | [0.29, 0.33] | 0.35 ± 0.08 | [0.32, 0.37] | -0.04 ± 0.06 | [-0.06, -0.01] | 0.00 |
|  |  | **LT** | 0.20 ± 0.04 | [0.19, 0.22] | 0.21 ± 0.05 | [0.20, 0.23] | -0.01 ± 0.04 | [-0.02, 0.00] | 0.12 |
|  |  | **cLF** | 0.30 ± 0.06 | [0.28, 0.32] | 0.32 ± 0.07 | [0.30, 0.34] | -0.02 ± 0.05 | [-0.04, 0.00] | 0.02 |

CL-JSN: radiographically normal knees with joint space narrowing (JSN) in the contralateral (CL) knee, CL-noROA: radiographically normal knees without signs of radiographic OA (ROA) also in the contralateral knee, SD: standard deviation, 95% CI: 95% confidence interval, Diff.: Mean difference, DSC: Dice Similarity Coefficient, HD: Hausdorff distance, ASSD: Average symmetric surface distance, VOE: volume overlap error, MT: medial tibia, cMF: central medial femur, LT: lateral tibia, cLF: central lateral femur, p-values for differences between the All_E_ and the 1^st^_E_ U-Net computed using paired t-tests.

**Supplemental Table 3: Agreement between re-trained U-Net-based and manual bone and cartilage segmentations from multi-echo spin-echo MRIs and comparison of the agreement between the re-trained U-Net trained from all echoes (All_E_) and the re-trained U-Net trained from the 1^st^ echo (1^st^_E_) in all knees with manual segmentations**

|  | | **All echoes U-Net** | | **First echo U-Net** | | **All echoes vs. 1^st^ echo U-Net** | | |
| --- | --- | --- | --- | --- | --- | --- | --- | --- |
|  |  | **Mean ± SD** | **[95% CI]** | **Mean ± SD** | **[95% CI]** | **Mean diff. ± SD** | **[95% CI]** | **p-value** |
| **Bone (HRC test set, n=10):** | | | | | | | |  |
| **DSC** | **Femur** | 0.98 ± 0.004 | [0.97, 0.98] | 0.98 ± 0.005 | [0.97, 0.98] | 0.00 ± 0.00 | [0.00, 0.00] | 0.03 |
|  | **Tibia** | 0.98 ± 0.003 | [0.98, 0.98] | 0.98 ± 0.005 | [0.97, 0.98] | 0.00 ± 0.00 | [0.00, 0.00] | 0.04 |
|  | **Fibula** | 0.94 ± 0.02 | [0.93, 0.96] | 0.93 ± 0.02 | [0.91, 0.95] | 0.01 ± 0.02 | [0.00, 0.03] | 0.04 |
| **HD (mm)** | **Femur** | 3.91 ± 0.44 | [3.59, 4.22] | 4.63 ± 1.36 | [3.66, 5.61] | -0.73 ± 1.26 | [-1.63, 0.18] | 0.10 |
|  | **Tibia** | 4.13 ± 1.22 | [3.26, 5.00] | 5.00 ± 3.84 | [2.25, 7.75] | -0.87 ± 2.66 | [-2.77, 1.03] | 0.33 |
|  | **Fibula** | 4.71 ± 1.26 | [3.81, 5.62] | 4.22 ± 0.85 | [3.61, 4.83] | 0.49 ± 1.19 | [-0.36, 1.35] | 0.22 |
| **ASSD (mm)** | **Femur** | 0.22 ± 0.06 | [0.18, 0.26] | 0.25 ± 0.07 | [0.20, 0.30] | -0.03 ± 0.05 | [-0.07, 0.01] | 0.09 |
|  | **Tibia** | 0.20 ± 0.08 | [0.15, 0.26] | 0.26 ± 0.18 | [0.13, 0.39] | -0.06 ± 0.13 | [-0.15, 0.03] | 0.19 |
|  | **Fibula** | 0.31 ± 0.22 | [0.15, 0.46] | 0.45 ± 0.28 | [0.25, 0.65] | -0.14 ± 0.22 | [-0.30, 0.01] | 0.07 |
| **VOE (%)** | **Femur** | 0.04 ± 0.01 | [0.04, 0.05] | 0.05 ± 0.01 | [0.04, 0.05] | 0.00 ± 0.00 | [-0.01, 0.00] | 0.03 |
|  | **Tibia** | 0.04 ± 0.01 | [0.04, 0.05] | 0.05 ± 0.01 | [0.04, 0.05] | 0.00 ± 0.01 | [-0.01, 0.00] | 0.04 |
|  | **Fibula** | 0.11 ± 0.03 | [0.08, 0.13] | 0.13 ± 0.04 | [0.10, 0.16] | -0.02 ± 0.03 | [-0.05, 0.00] | 0.04 |
| **Cartilage (HRC test set, matched cases and controls, n=88):** | | | | | | | |  |
| **DSC** | **MT** | 0.87 ± 0.03 | [0.86, 0.87] | 0.86 ± 0.03 | [0.86, 0.87] | 0.00 ± 0.02 | [0.00, 0.01] | 0.09 |
|  | **cMF** | 0.81 ± 0.04 | [0.80, 0.82] | 0.78 ± 0.06 | [0.77, 0.79] | 0.03 ± 0.05 | [0.02, 0.04] | <0.01 |
|  | **LT** | 0.88 ± 0.03 | [0.87, 0.89] | 0.88 ± 0.03 | [0.87, 0.89] | 0.00 ± 0.02 | [0.00, 0.01] | 0.33 |
|  | **cLF** | 0.83 ± 0.04 | [0.82, 0.84] | 0.81 ± 0.05 | [0.80, 0.82] | 0.02 ± 0.04 | [0.01, 0.03] | <0.01 |
| **HD (mm)** | **MT** | 4.28 ± 1.47 | [3.97, 4.59] | 3.95 ± 1.08 | [3.72, 4.17] | 0.33 ± 1.25 | [0.07, 0.60] | 0.01 |
|  | **cMF** | 4.63 ± 1.82 | [4.24, 5.01] | 4.83 ± 1.56 | [4.50, 5.16] | -0.20 ± 1.88 | [-0.60, 0.19] | 0.31 |
|  | **LT** | 4.91 ± 4.19 | [4.02, 5.80] | 4.73 ± 4.37 | [3.81, 5.66] | 0.18 ± 1.31 | [-0.10, 0.45] | 0.21 |
|  | **cLF** | 4.22 ± 1.54 | [3.89, 4.54] | 4.61 ± 1.63 | [4.27, 4.96] | -0.40 ± 1.64 | [-0.74, -0.05] | 0.03 |
| **ASSD (mm)** | **MT** | 0.19 ± 0.16 | [0.16, 0.22] | 0.18 ± 0.13 | [0.15, 0.21] | 0.01 ± 0.09 | [-0.01, 0.03] | 0.21 |
|  | **cMF** | 0.32 ± 0.22 | [0.27, 0.37] | 0.40 ± 0.22 | [0.35, 0.44] | -0.08 ± 0.25 | [-0.13, -0.02] | 0.01 |
|  | **LT** | 0.19 ± 0.11 | [0.17, 0.22] | 0.20 ± 0.10 | [0.18, 0.22] | 0.00 ± 0.08 | [-0.02, 0.01] | 0.64 |
|  | **cLF** | 0.21 ± 0.14 | [0.18, 0.24] | 0.27 ± 0.22 | [0.22, 0.32] | -0.06 ± 0.19 | [-0.10, -0.02] | 0.01 |
| **VOE (%)** | **MT** | 0.23 ± 0.05 | [0.22, 0.24] | 0.24 ± 0.05 | [0.23, 0.25] | -0.01 ± 0.03 | [-0.01, 0.00] | 0.08 |
|  | **cMF** | 0.31 ± 0.06 | [0.30, 0.32] | 0.36 ± 0.08 | [0.34, 0.37] | -0.04 ± 0.07 | [-0.06, -0.03] | <0.01 |
|  | **LT** | 0.21 ± 0.05 | [0.20, 0.22] | 0.22 ± 0.05 | [0.21, 0.23] | 0.00 ± 0.03 | [-0.01, 0.00] | 0.34 |
|  | **cLF** | 0.28 ± 0.06 | [0.27, 0.30] | 0.31 ± 0.07 | [0.30, 0.33] | -0.03 ± 0.05 | [-0.04, -0.02] | <0.01 |

SD: standard deviation, 95% CI: 95% confidence interval, Diff.: Mean difference, HRC: Healthy reference cohort, CL-JSN: radiographically normal knees with joint space narrowing (JSN) in the contralateral (CL) knee, CL-noROA: radiographically normal knees without signs of radiographic OA (ROA) also in the contralateral knee, DSC: Dice Similarity Coefficient, HD: Hausdorff distance, ASSD: Average symmetric surface distance, VOE: volume overlap error, MT: medial tibia, cMF: central medial femur, LT: lateral tibia, cLF: central lateral femur, p-values for differences between the All_E_ and the 1^st^_E_ U-Net computed using paired t-tests.

**Supplemental Table 4: Comparison of superficial and deep layer T2 times (in ms) between manual cartilage segmentations and U-Net-based cartilage segmentations (all echoes (All_E_) U-Net & 1^st^ echo (1^st^_E_) U-Net) in healthy reference cohort (HRC) test set, the 39 CL-JSN, and the 39 matched CL-noROA knees**

|  | | **Manual** | **All_E_ U-Net** | | | | | **1^st^_E_ U-Net** | | | | |
| --- | --- | --- | --- | --- | --- | --- | --- | --- | --- | --- | --- | --- |
|  |  | **Mean±SD** | **Mean±SD** | **Diff. ± SD** | **95% CI** | **p-value** | **r** | **Mean±SD** | **Diff. ± SD** | **95% CI** | **p-value** | **r** |
| **HRC test set (n=10):** | | | | | | | | | | | | |
| **Deep layer** | **FTJ** | 35.0± 2.5 | 34.5± 1.7 | -0.6± 1.2 | [-1.4, 0.3] | 0.17 | 0.89 | 35.0± 1.8 | 0.0± 1.0 | [-0.7, 0.7] | 0.95 | 0.93 |
|  | **MFTC** | 35.2± 2.7 | 34.6± 2.0 | -0.6± 1.4 | [-1.6, 0.5] | 0.25 | 0.86 | 35.3± 1.9 | 0.1± 1.5 | [-0.9, 1.2] | 0.76 | 0.86 |
|  | **LFTC** | 34.9± 2.4 | 34.3± 1.8 | -0.6± 1.1 | [-1.3, 0.2] | 0.13 | 0.92 | 34.7± 2.1 | -0.2± 0.8 | [-0.7, 0.4] | 0.47 | 0.96 |
|  | **MT** | 32.6± 2.3 | 32.6± 1.9 | 0.0± 0.6 | [-0.4, 0.4] | 0.99 | 0.97 | 33.4± 1.9 | 0.8± 0.8 | [0.3, 1.4] | 0.01 | 0.94 |
|  | **cMF** | 37.8± 3.9 | 36.7± 2.5 | -1.1± 2.6 | [-3.0, 0.8] | 0.21 | 0.74 | 37.3± 2.6 | -0.6± 2.7 | [-2.5, 1.4] | 0.54 | 0.72 |
|  | **LT** | 31.5± 2.2 | 31.2± 2.0 | -0.2± 0.6 | [-0.7, 0.2] | 0.23 | 0.96 | 31.8± 2.0 | 0.3± 0.4 | [0.0, 0.5] | 0.04 | 0.99 |
|  | **cLF** | 38.3± 3.1 | 37.4± 1.9 | -0.9± 1.7 | [-2.1, 0.3] | 0.12 | 0.89 | 37.6± 2.5 | -0.6± 1.4 | [-1.6, 0.3] | 0.18 | 0.91 |
| **Superficial layer** | **FTJ** | 44.3± 3.4 | 45.0± 3.3 | 0.7± 0.7 | [0.2, 1.2] | 0.01 | 0.98 | 45.4± 3.2 | 1.1± 1.0 | [0.4, 1.8] | 0.01 | 0.96 |
|  | **MFTC** | 44.3± 4.0 | 45.4± 3.9 | 1.1± 1.2 | [0.2, 2.0] | 0.02 | 0.95 | 46.2± 3.8 | 1.9± 1.7 | [0.7, 3.1] | 0.01 | 0.91 |
|  | **LFTC** | 44.3± 3.1 | 44.6± 3.1 | 0.3± 0.3 | [0.1, 0.5] | 0.02 | 0.99 | 44.6± 3.1 | 0.3± 0.9 | [-0.3, 0.9] | 0.34 | 0.96 |
|  | **MT** | 40.7± 3.4 | 42.0± 3.3 | 1.3± 1.4 | [0.3, 2.3] | 0.02 | 0.91 | 41.9± 3.4 | 1.2± 1.5 | [0.1, 2.3] | 0.03 | 0.90 |
|  | **cMF** | 47.9± 4.9 | 48.8± 4.7 | 0.9± 1.7 | [-0.3, 2.1] | 0.12 | 0.94 | 50.5± 4.7 | 2.6± 2.8 | [0.6, 4.6] | 0.02 | 0.83 |
|  | **LT** | 42.3± 3.2 | 42.2± 3.4 | -0.1± 0.9 | [-0.8, 0.5] | 0.70 | 0.96 | 42.3± 3.1 | -0.1± 1.3 | [-1.0, 0.9] | 0.91 | 0.91 |
|  | **cLF** | 46.3± 3.6 | 47.0± 3.1 | 0.7± 0.8 | [0.1, 1.3] | 0.02 | 0.98 | 46.9± 3.3 | 0.6± 1.1 | [-0.2, 1.4] | 0.10 | 0.95 |
| **All CL-JSN knees (n=39):** | | | | | | | | | | | | |
| **Deep layer** | **FTJ** | 37.8± 2.3 | 36.7± 2.0 | -1.2± 0.8 | [-1.4, -0.9] | <0.01 | 0.91 | 38.1± 2.7 | 0.2± 1.5 | [-0.2, 0.7] | 0.31 | 0.80 |
|  | **MFTC** | 38.4± 2.6 | 37.1± 2.3 | -1.2± 1.1 | [-1.6, -0.9] | <0.01 | 0.91 | 39.1± 3.6 | 0.8± 2.9 | [-0.2, 1.7] | 0.11 | 0.77 |
|  | **LFTC** | 37.3± 2.6 | 36.2± 2.2 | -1.1± 0.9 | [-1.4, -0.8] | <0.01 | 0.88 | 37.1± 2.5 | -0.3± 0.9 | [-0.5, 0.0] | 0.09 | 0.79 |
|  | **MT** | 33.9± 2.1 | 33.6± 2.0 | -0.2± 0.5 | [-0.4, -0.1] | 0.01 | 0.87 | 34.1± 2.0 | 0.2± 0.6 | [0.1, 0.4] | 0.01 | 0.79 |
|  | **cMF** | 42.8± 4.0 | 40.6± 3.3 | -2.2± 2.4 | [-3.0, -1.4] | <0.01 | 0.91 | 44.1± 6.9 | 1.3± 5.8 | [-0.6, 3.1] | 0.18 | 0.77 |
|  | **LT** | 32.9± 2.3 | 32.2± 2.0 | -0.7± 0.7 | [-0.9, -0.5] | <0.01 | 0.89 | 32.9± 2.5 | 0.0± 0.9 | [-0.3, 0.3] | 0.84 | 0.79 |
|  | **cLF** | 41.8± 3.9 | 40.2± 3.3 | -1.6± 1.3 | [-2.0, -1.2] | <0.01 | 0.85 | 41.3± 3.8 | -0.5± 1.4 | [-0.9, 0.0] | 0.04 | 0.78 |
| **Superficial layer** | **FTJ** | 49.0± 3.8 | 48.3± 3.2 | -0.7± 1.6 | [-1.2, -0.1] | 0.01 | 0.90 | 51.4± 6.3 | 2.4± 4.0 | [1.1, 3.7] | <0.01 | 0.61 |
|  | **MFTC** | 49.6± 4.7 | 48.9± 3.6 | -0.7± 2.1 | [-1.4, 0.0] | 0.04 | 0.95 | 53.2± 10.4 | 3.6± 7.4 | [1.2, 6.0] | <0.01 | 0.94 |
|  | **LFTC** | 48.4± 3.7 | 47.7± 3.4 | -0.6± 1.8 | [-1.2, -0.1] | 0.03 | 0.97 | 49.6± 4.7 | 1.2± 2.9 | [0.3, 2.1] | 0.01 | 0.96 |
|  | **MT** | 44.4± 3.2 | 44.4± 2.5 | 0.0± 1.6 | [-0.5, 0.5] | 0.90 | 0.80 | 44.8± 3.6 | 0.4± 2.3 | [-0.3, 1.2] | 0.25 | 0.54 |
|  | **cMF** | 54.8± 6.8 | 53.4± 5.2 | -1.4± 3.1 | [-2.4, -0.4] | 0.01 | 0.96 | 61.5± 18.1 | 6.7± 13.6 | [2.3, 11.1] | <0.01 | 0.93 |
|  | **LT** | 45.6± 3.4 | 44.8± 3.0 | -0.7± 1.5 | [-1.2, -0.2] | <0.01 | 0.94 | 46.0± 4.4 | 0.4± 2.7 | [-0.4, 1.3] | 0.31 | 0.94 |
|  | **cLF** | 51.2± 4.6 | 50.7± 4.4 | -0.6± 2.5 | [-1.4, 0.2] | 0.17 | 0.94 | 53.2± 6.1 | 1.9± 3.8 | [0.7, 3.2] | <0.01 | 0.94 |
| **All CL-noROA knees (n=39):** | | | | | | | | | | | | |
| **Deep layer** | **FTJ** | 37.1± 2.0 | 35.8± 1.7 | -1.3± 0.9 | [-1.6, -1.1] | <0.01 | 0.76 | 36.6± 1.8 | -0.5± 1.0 | [-0.9, -0.2] | <0.01 | 0.74 |
|  | **MFTC** | 37.7± 2.4 | 36.3± 1.9 | -1.4± 1.4 | [-1.8, -0.9] | <0.01 | 0.82 | 37.2± 2.3 | -0.5± 1.7 | [-1.1, 0.0] | 0.07 | 0.75 |
|  | **LFTC** | 36.5± 2.3 | 35.2± 1.9 | -1.3± 0.9 | [-1.6, -1.0] | <0.01 | 0.75 | 35.9± 2.2 | -0.6± 0.9 | [-0.9, -0.3] | <0.01 | 0.69 |
|  | **MT** | 33.4± 2.1 | 33.1± 1.9 | -0.3± 0.6 | [-0.5, -0.1] | <0.01 | 0.85 | 33.6± 1.9 | 0.2± 0.7 | [0.0, 0.4] | 0.08 | 0.72 |
|  | **cMF** | 42.1± 4.1 | 39.6± 2.8 | -2.4± 2.8 | [-3.4, -1.5] | <0.01 | 0.75 | 40.9± 4.2 | -1.2± 3.3 | [-2.3, -0.1] | 0.03 | 0.69 |
|  | **LT** | 32.0± 2.1 | 31.3± 1.8 | -0.7± 0.9 | [-1.0, -0.5] | <0.01 | 0.67 | 31.8± 2.0 | -0.3± 0.9 | [-0.6, 0.0] | 0.07 | 0.71 |
|  | **cLF** | 41.0± 3.3 | 39.1± 2.9 | -1.9± 1.4 | [-2.3, -1.4] | <0.01 | 0.84 | 40.0± 3.3 | -0.9± 1.5 | [-1.4, -0.4] | <0.01 | 0.71 |
| **Superficial layer** | **FTJ** | 47.2± 2.8 | 46.5± 2.4 | -0.7± 1.8 | [-1.3, -0.1] | 0.02 | 0.82 | 48.0± 3.6 | 0.8± 2.4 | [0.0, 1.6] | 0.05 | 0.74 |
|  | **MFTC** | 47.6± 4.0 | 46.9± 2.7 | -0.7± 2.3 | [-1.5, 0.0] | 0.06 | 0.92 | 48.6± 5.7 | 1.0± 3.8 | [-0.2, 2.2] | 0.10 | 0.92 |
|  | **LFTC** | 46.8± 2.7 | 46.1± 2.9 | -0.7± 2.0 | [-1.3, -0.1] | 0.03 | 0.96 | 47.4± 3.9 | 0.5± 2.9 | [-0.4, 1.5] | 0.25 | 0.94 |
|  | **MT** | 43.2± 3.1 | 43.0± 2.5 | -0.1± 1.6 | [-0.7, 0.4] | 0.57 | 0.72 | 43.3± 3.7 | 0.2± 2.6 | [-0.7, 1.0] | 0.72 | 0.68 |
|  | **cMF** | 52.0± 5.6 | 50.8± 3.8 | -1.3± 3.8 | [-2.5, 0.0] | 0.04 | 0.91 | 53.9± 8.8 | 1.9± 6.4 | [-0.2, 4.0] | 0.07 | 0.90 |
|  | **LT** | 44.2± 3.1 | 43.6± 2.8 | -0.6± 2.4 | [-1.4, 0.2] | 0.12 | 0.91 | 43.8± 3.5 | -0.3± 2.5 | [-1.2, 0.5] | 0.41 | 0.90 |
|  | **cLF** | 49.5± 3.4 | 48.7± 3.9 | -0.8± 2.1 | [-1.5, -0.1] | 0.02 | 0.97 | 50.9± 5.5 | 1.4± 3.9 | [0.1, 2.7] | 0.03 | 0.98 |

CL-JSN: radiographically normal knees with joint space narrowing (JSN) in the contralateral (CL) knee, CL-noROA: radiographically normal knees without signs of radiographic OA (ROA) also in the contralateral knee, SD: standard deviation, 95% CI: 95% confidence interval, r: Pearson correlation coefficient, Diff.: Mean difference, FTJ: Femorotibial joint, MFTC: medial femorotibial compartment, LFTC: lateral femorotibial compartment, MT: medial tibia, cMF: central medial femur, LT: lateral tibia, cLF: central lateral femur, p-values for differences between U-Net-based and manual cartilage T2 computed using from paired t-tests

**Supplemental Table 5: Comparison of superficial and deep layer T2 times between manual cartilage segmentations and U-Net-based cartilage segmentations from re-trained U-Nets (all echoes (All_E_) U-Net & 1^st^ echo (1^st^_E_) U-Net) in all 88 knees with manual segmentations (healthy reference cohort test set: n=10, CL-JSN: n=39, CL-noROA: n=39)**

|  | **Manual** | **All echoes U-Net** | | | | | **1^st^ echo U-Net** | | | | |
| --- | --- | --- | --- | --- | --- | --- | --- | --- | --- | --- | --- |
|  | **Mean ± SD** | **Mean ± SD** | **Mean Diff ± SD** | **[95% CI]** | **p-value** | **r** | **Mean ± SD** | **Mean Diff ± SD** | **[95% CI]** | **p-value** | **r** |
| **Deep layer:** | | | | | | | | | | | |
| **FTJ** | 37.4 ± 2.1 | 36.3 ± 1.8 | -1.1 ± 0.9 | [-1.3, -0.9] | <0.01 | 0.91 | 37.9 ± 2.5 | 0.5 ± 1.5 | [0.2, 0.8] | <0.01 | 0.81 |
| **MFTC** | 38.0 ± 2.4 | 36.7 ± 2.0 | -1.2 ± 1.2 | [-1.5, -1.0] | <0.01 | 0.86 | 39.0 ± 3.4 | 1.0 ± 2.6 | [0.5, 1.6] | <0.01 | 0.64 |
| **LFTC** | 36.8 ± 2.4 | 35.9 ± 2.1 | -0.9 ± 1.0 | [-1.1, -0.7] | <0.01 | 0.92 | 36.7 ± 2.4 | 0.0 ± 1.2 | [-0.3, 0.2] | 0.73 | 0.89 |
| **MT** | 33.6 ± 2.1 | 33.4 ± 2.0 | -0.2 ± 0.6 | [-0.3, -0.1] | <0.01 | 0.96 | 33.8 ± 2.1 | 0.2 ± 0.7 | [0.0, 0.3] | 0.02 | 0.94 |
| **cMF** | 42.3 ± 3.9 | 40.0 ± 3.0 | -2.3 ± 2.5 | [-2.8, -1.7] | <0.01 | 0.77 | 44.1 ± 6.3 | 1.8 ± 5.2 | [0.7, 2.9] | <0.01 | 0.57 |
| **LT** | 32.3 ± 2.2 | 31.8 ± 1.9 | -0.6 ± 0.8 | [-0.7, -0.4] | <0.01 | 0.93 | 32.3 ± 2.2 | 0.0 ± 1.0 | [-0.2, 0.2] | 0.94 | 0.90 |
| **cLF** | 41.3 ± 3.5 | 40.0 ± 3.1 | -1.3 ± 1.4 | [-1.6, -1.0] | <0.01 | 0.92 | 41.2 ± 3.8 | -0.1 ± 1.8 | [-0.5, 0.3] | 0.63 | 0.87 |
| **Superficial layer:** | | | | | | | | | | | |
| **FTJ** | 48.0 ± 3.3 | 47.9 ± 3.0 | -0.1 ± 1.8 | [-0.5, 0.3] | 0.68 | 0.83 | 51.7 ± 5.7 | 3.8 ± 4.0 | [2.9, 4.6] | <0.01 | 0.74 |
| **MFTC** | 48.5 ± 4.3 | 48.4 ± 3.6 | -0.1 ± 2.2 | [-0.5, 0.4] | 0.77 | 0.85 | 53.8 ± 9.2 | 5.3 ± 6.7 | [3.9, 6.7] | <0.01 | 0.74 |
| **LFTC** | 47.5 ± 3.2 | 47.4 ± 3.1 | -0.1 ± 2.0 | [-0.5, 0.3] | 0.67 | 0.79 | 49.7 ± 4.8 | 2.2 ± 3.5 | [1.5, 3.0] | <0.01 | 0.67 |
| **MT** | 43.7 ± 3.1 | 43.6 ± 2.6 | -0.1 ± 1.7 | [-0.4, 0.3] | 0.75 | 0.84 | 46.8 ± 5.0 | 3.2 ± 3.5 | [2.4, 3.9] | <0.01 | 0.73 |
| **cMF** | 53.3 ± 6.1 | 53.2 ± 5.4 | -0.1 ± 3.4 | [-0.8, 0.6] | 0.82 | 0.83 | 60.7 ± 15.0 | 7.5 ± 11.4 | [5.0, 9.9] | <0.01 | 0.72 |
| **LT** | 44.7 ± 3.1 | 43.8 ± 2.9 | -0.9 ± 2.0 | [-1.3, -0.5] | <0.01 | 0.78 | 45.9 ± 4.3 | 1.2 ± 3.0 | [0.5, 1.8] | <0.01 | 0.71 |
| **cLF** | 50.3 ± 3.9 | 51.0 ± 4.2 | 0.7 ± 2.6 | [0.2, 1.3] | 0.01 | 0.80 | 53.5 ± 6.4 | 3.2 ± 4.9 | [2.2, 4.3] | <0.01 | 0.64 |

CL-JSN: radiographically normal knees with joint space narrowing (JSN) in the contralateral (CL) knee, CL-noROA: radiographically normal knees without signs of radiographic OA (ROA) also in the contralateral knee, SD: standard deviation, 95% CI: 95% confidence interval, r: Pearson correlation coefficient, Diff.: Mean difference, FTJ: Femorotibial joint, MFTC: medial femorotibial compartment, LFTC: lateral femorotibial compartment, MT: medial tibia, cMF: central medial femur, LT: lateral tibia, cLF: central lateral femur, p-values for differences between U-Net-based and manual cartilage T2 computed using paired t-tests

**Supplemental Table 6: Comparison of superficial and deep layer T2 times at the year 1 visit (in ms) in cartilage plates from manual and all echoes (All_E_) U-Net segmentations between matched CL-JSN (n=39) and CL-noROA knees (n=39) and between CL-JSN (n=39) and non-matched CL-noROA knees (n=982)**

|  | | **CL-JSN** | **CL-noROA** | **CL-JSN vs CL-noROA** | | | | |
| --- | --- | --- | --- | --- | --- | --- | --- | --- |
|  |  | **Mean ± SD** | **Mean ± SD** | **Mean diff.** | **[95% CI]** | **Cohen's D** | **[95% CI]** | **p-value** |
| **Matched comparison, manual segmentation:** | | | | | | | | |
| **Deep layer** | **MT** | 33.9 ± 2.1 | 33.4 ± 2.1 | 0.5 | [-0.5, 1.5] | 0.16 | [-0.16, 0.48] | 0.32 |
|  | **cMF** | 42.8 ± 4.0 | 42.1 ± 4.1 | 0.8 | [-0.8, 2.3] | 0.16 | [-0.16, 0.47] | 0.33 |
|  | **LT** | 32.9 ± 2.3 | 32.0 ± 2.1 | 0.9 | [-0.2, 1.9] | 0.28 | [-0.05, 0.59] | 0.09 |
|  | **cLF** | 41.8 ± 3.9 | 41.0 ± 3.3 | 0.8 | [-0.7, 2.3] | 0.18 | [-0.14, 0.49] | 0.27 |
| **Superficial layer** | **MT** | 44.4 ± 3.2 | 43.2 ± 3.1 | 1.2 | [-0.1, 2.5] | 0.30 | [-0.02, 0.62] | 0.07 |
|  | **cMF** | 54.8 ± 6.8 | 52.0 ± 5.6 | 2.8 | [0.6, 5.0] | 0.41 | [0.08, 0.73] | 0.02 |
|  | **LT** | 45.6 ± 3.4 | 44.2 ± 3.1 | 1.4 | [0.1, 2.7] | 0.35 | [0.03, 0.68] | 0.03 |
|  | **cLF** | 51.2 ± 4.6 | 49.5 ± 3.4 | 1.7 | [0.1, 3.3] | 0.34 | [0.02, 0.66] | 0.04 |
| **Matched comparison, All_E_ U-Net:** | | | | | | | | |
| **Deep layer** | **MT** | 33.6 ± 2.0 | 33.1 ± 1.9 | 0.6 | [-0.4, 1.5] | 0.20 | [-0.12, 0.52] | 0.22 |
|  | **cMF** | 40.6 ± 3.3 | 39.6 ± 2.8 | 1.0 | [-0.1, 2.1] | 0.29 | [-0.03, 0.61] | 0.08 |
|  | **LT** | 32.2 ± 2.0 | 31.3 ± 1.8 | 1.0 | [0.1, 1.8] | 0.36 | [0.03, 0.68] | 0.03 |
|  | **cLF** | 40.2 ± 3.3 | 39.1 ± 2.9 | 1.1 | [-0.1, 2.3] | 0.30 | [-0.03, 0.62] | 0.07 |
| **Superficial layer** | **MT** | 44.4 ± 2.5 | 43.0 ± 2.5 | 1.4 | [0.3, 2.5] | 0.42 | [0.09, 0.74] | 0.01 |
|  | **cMF** | 53.4 ± 5.2 | 50.8 ± 3.8 | 2.6 | [1.1, 4.2] | 0.55 | [0.21, 0.88] | <0.01 |
|  | **LT** | 44.8 ± 3.0 | 43.6 ± 2.8 | 1.3 | [0.0, 2.6] | 0.32 | [0.00, 0.64] | 0.05 |
|  | **cLF** | 50.7 ± 4.4 | 48.7 ± 3.9 | 1.9 | [0.3, 3.5] | 0.39 | [0.06, 0.72] | 0.02 |
| **Non-matched comparison, All_E_ U-Net:** | | | | | | | | |
| **Deep layer** | **MT** | 33.6± 2.0 | 33.0± 2.0 | 0.6 | [0.0, 1.2] | 0.30 | [-0.02, 0.62] | 0.06 |
|  | **cMF** | 40.6± 3.3 | 40.2± 3.3 | 0.5 | [-0.6, 1.5] | 0.14 | [-0.18, 0.46] | 0.40 |
|  | **LT** | 32.2± 2.0 | 31.3± 2.1 | 0.9 | [0.3, 1.6] | 0.45 | [0.13, 0.77] | 0.01 |
|  | **cLF** | 40.2± 3.3 | 39.8± 2.9 | 0.3 | [-0.6, 1.3] | 0.12 | [-0.20, 0.44] | 0.47 |
| **Superficial layer** | **MT** | 44.4± 2.5 | 43.0± 2.8 | 1.4 | [0.5, 2.3] | 0.50 | [0.17, 0.82] | <0.01 |
|  | **cMF** | 53.4± 5.2 | 51.6± 4.2 | 1.7 | [0.4, 3.1] | 0.41 | [0.09, 0.73] | 0.01 |
|  | **LT** | 44.8± 3.0 | 43.4± 2.9 | 1.5 | [0.6, 2.4] | 0.51 | [0.19, 0.83] | <0.01 |
|  | **cLF** | 50.7± 4.4 | 49.4± 3.5 | 1.3 | [0.1, 2.4] | 0.36 | [0.04, 0.68] | 0.03 |

CL-JSN: radiographically normal knees with joint space narrowing (JSN) in the contralateral (CL) knee, CL-noROA: radiographically normal knees without signs of radiographic OA (ROA) also in the contralateral knee, SD: standard deviation, 95% CI: 95% confidence interval, r: Pearson correlation coefficient, MT: medial tibia, cMF: central medial femur, LT: lateral tibia, cLF: central lateral femur, Mean diff.: Mean difference, p-values for differences between CL-JSN and CL-noROA knees computed using paired (matched comparisons) or unpaired t-tests (non-matched comparison)

**Supplemental Table 7: Comparison of superficial and deep layer T2 change between the year-1 and -4 visits (in ms) in cartilage plates from manual and all echoes (All_E_) U-Net segmentations between matched CL-JSN (n=39) and CL-noROA knees (n=39) and between CL-JSN (n=39) and non-matched CL-noROA knees (n=777)**

|  | | **CL-JSN** | **CL-noROA** | **CL-JSN vs CL-noROA** | | | | |
| --- | --- | --- | --- | --- | --- | --- | --- | --- |
|  |  | **Mean ± SD** | **Mean ± SD** | **Mean diff.** | **[95% CI]** | **Cohen's D** | **[95% CI]** | **p-value** |
| **Matched comparison, manual segmentation:** | | | | | | | | |
| **Deep layer** | **MT** | 1.1 ± 2.3 | 0.6 ± 2.4 | 0.6 | [-0.5, 1.6] | 0.17 | [-0.14, 0.49] | 0.28 |
|  | **cMF** | 1.6 ± 5.3 | 0.2 ± 3.1 | 1.4 | [-0.5, 3.4] | 0.24 | [-0.08, 0.55] | 0.15 |
|  | **LT** | 1.5 ± 2.2 | 0.2 ± 2.0 | 1.3 | [0.4, 2.3] | 0.45 | [0.12, 0.78] | 0.01 |
|  | **cLF** | 0.5 ± 3.2 | -0.5 ± 2.0 | 1.0 | [-0.1, 2.1] | 0.30 | [-0.03, 0.62] | 0.07 |
| **Superficial layer** | **MT** | 0.8 ± 2.5 | 1.1 ± 2.3 | -0.3 | [-1.3, 0.7] | -0.09 | [-0.41, 0.22] | 0.56 |
|  | **cMF** | 1.8 ± 3.2 | 1.5 ± 2.9 | 0.3 | [-1.1, 1.7] | 0.08 | [-0.24, 0.39] | 0.63 |
|  | **LT** | 1.0 ± 1.7 | 0.7 ± 2.4 | 0.3 | [-0.8, 1.5] | 0.10 | [-0.22, 0.41] | 0.53 |
|  | **cLF** | 0.0 ± 3.2 | 0.3 ± 1.9 | -0.3 | [-1.6, 1.0] | -0.08 | [-0.39, 0.24] | 0.62 |
| **Matched comparison, All_E_ U-Net:** | | | | | | | | |
| **Deep layer** | **MT** | 1.0 ± 2.1 | 0.1 ± 2.3 | 0.8 | [-0.1, 1.8] | 0.29 | [-0.03, 0.61] | 0.08 |
|  | **cMF** | 0.7 ± 2.5 | -0.1 ± 2.1 | 0.8 | [-0.3, 1.9] | 0.24 | [-0.08, 0.56] | 0.14 |
|  | **LT** | 1.1 ± 1.8 | -0.2 ± 1.9 | 1.3 | [0.5, 2.1] | 0.50 | [0.16, 0.83] | <0.01 |
|  | **cLF** | 0.5 ± 2.7 | -0.9 ± 1.7 | 1.4 | [0.3, 2.4] | 0.42 | [0.09, 0.75] | 0.01 |
| **Superficial layer** | **MT** | 0.5 ± 1.4 | 0.6 ± 1.8 | -0.2 | [-0.9, 0.6] | -0.07 | [-0.39, 0.24] | 0.66 |
|  | **cMF** | 0.9 ± 2.3 | 1.1 ± 2.6 | -0.2 | [-1.2, 0.8] | -0.06 | [-0.37, 0.26] | 0.72 |
|  | **LT** | 0.6 ± 1.7 | 0.2 ± 1.3 | 0.4 | [-0.3, 1.1] | 0.17 | [-0.15, 0.48] | 0.30 |
|  | **cLF** | 0.1 ± 2.4 | 0.0 ± 2.0 | 0.1 | [-1.0, 1.2] | 0.04 | [-0.27, 0.35] | 0.81 |
| **Non-matched comparison, All_E_ U-Net:** | | | | | | | | |
| **Deep layer** | **MT** | 1.0± 2.1 | 0.5± 2.2 | 0.4 | [-0.3, 1.1] | 0.19 | [-0.13, 0.51] | 0.25 |
|  | **cMF** | 0.7± 2.5 | 0.3± 2.5 | 0.4 | [-0.4, 1.1] | 0.14 | [-0.18, 0.46] | 0.39 |
|  | **LT** | 1.1± 1.8 | 0.3± 1.9 | 0.8 | [0.2, 1.4] | 0.43 | [0.11, 0.75] | 0.01 |
|  | **cLF** | 0.5± 2.7 | -0.1± 2.1 | 0.6 | [-0.1, 1.3] | 0.27 | [-0.05, 0.59] | 0.10 |
| **Superficial layer** | **MT** | 0.5± 1.4 | 0.5± 1.7 | 0.0 | [-0.6, 0.6] | 0.00 | [-0.32, 0.33] | 0.98 |
|  | **cMF** | 0.9± 2.3 | 0.9± 2.4 | 0.0 | [-0.8, 0.7] | -0.02 | [-0.34, 0.30] | 0.91 |
|  | **LT** | 0.6± 1.7 | 0.3± 1.4 | 0.2 | [-0.2, 0.7] | 0.16 | [-0.16, 0.49] | 0.32 |
|  | **cLF** | 0.1± 2.4 | 0.3± 1.8 | -0.2 | [-0.8, 0.4] | -0.12 | [-0.44, 0.20] | 0.45 |

CL-JSN: radiographically normal knees with joint space narrowing (JSN) in the contralateral (CL) knee, CL-noROA: radiographically normal knees without signs of radiographic OA (ROA) also in the contralateral knee, SD: standard deviation, 95% CI: 95% confidence interval, r: Pearson correlation coefficient, MT: medial tibia, cMF: central medial femur, LT: lateral tibia, cLF: central lateral femur, Diff.: Mean difference, p-values for differences between CL-JSN and CL-noROA knees computed using paired (matched comparisons) or unpaired t-tests (non-matched comparison)

**Supplemental Figure 1:** Illustration of the landmarks used for defining the femoral region of interest (ROI) and the fibula position, which allows to distinguish between the medial and the lateral femorotibial compartment. The arrows indicate the points used to define the femoral ROI: The most posterior points of the lateral and medial femoral condyle and the trochlea of the femur. The central (weight-bearing) part of the femoral cartilages is defined as the area between the anterior limitation (orange lines) and the plane at 75% of the anterior to posterior distance (green lines).


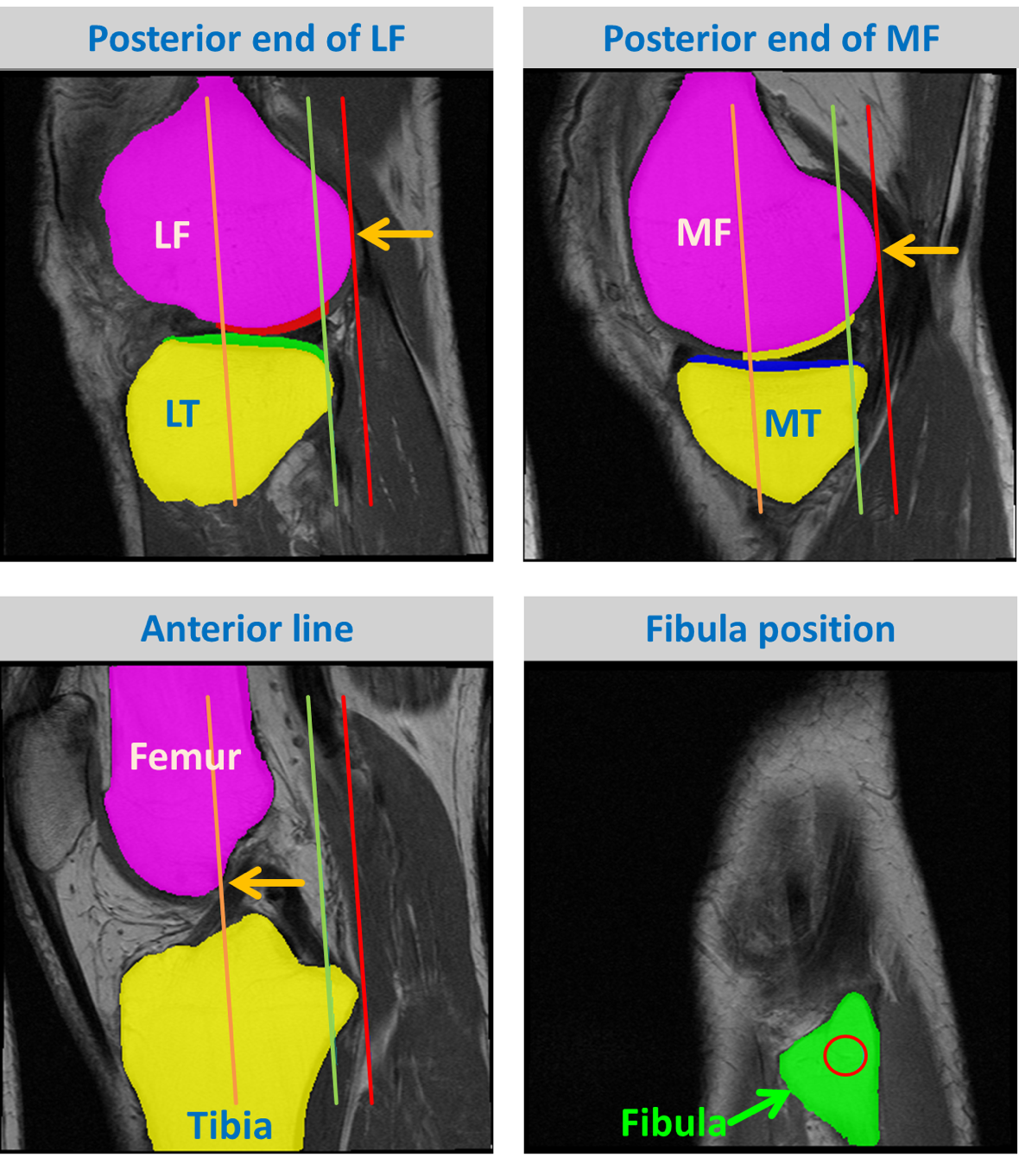


**Supplemental Figure 2:** Bland Altman plots visualizing the agreement of laminar T2 times in the medial and lateral femorotibial compartment computed from U-Net-based cartilage segmentations and manual cartilage segmentations. The first/second row shows the agreement for the All_E_ U-Net for the medial/lateral femorotibial compartment, the third/fourth row shows the agreement for the 1^st^_E_ U-Net for the medial/lateral femorotibial compartment. The left column shows the agreement for the deep, the right column shows the agreement for the superficial layer. Please note that the scales differ between plots.


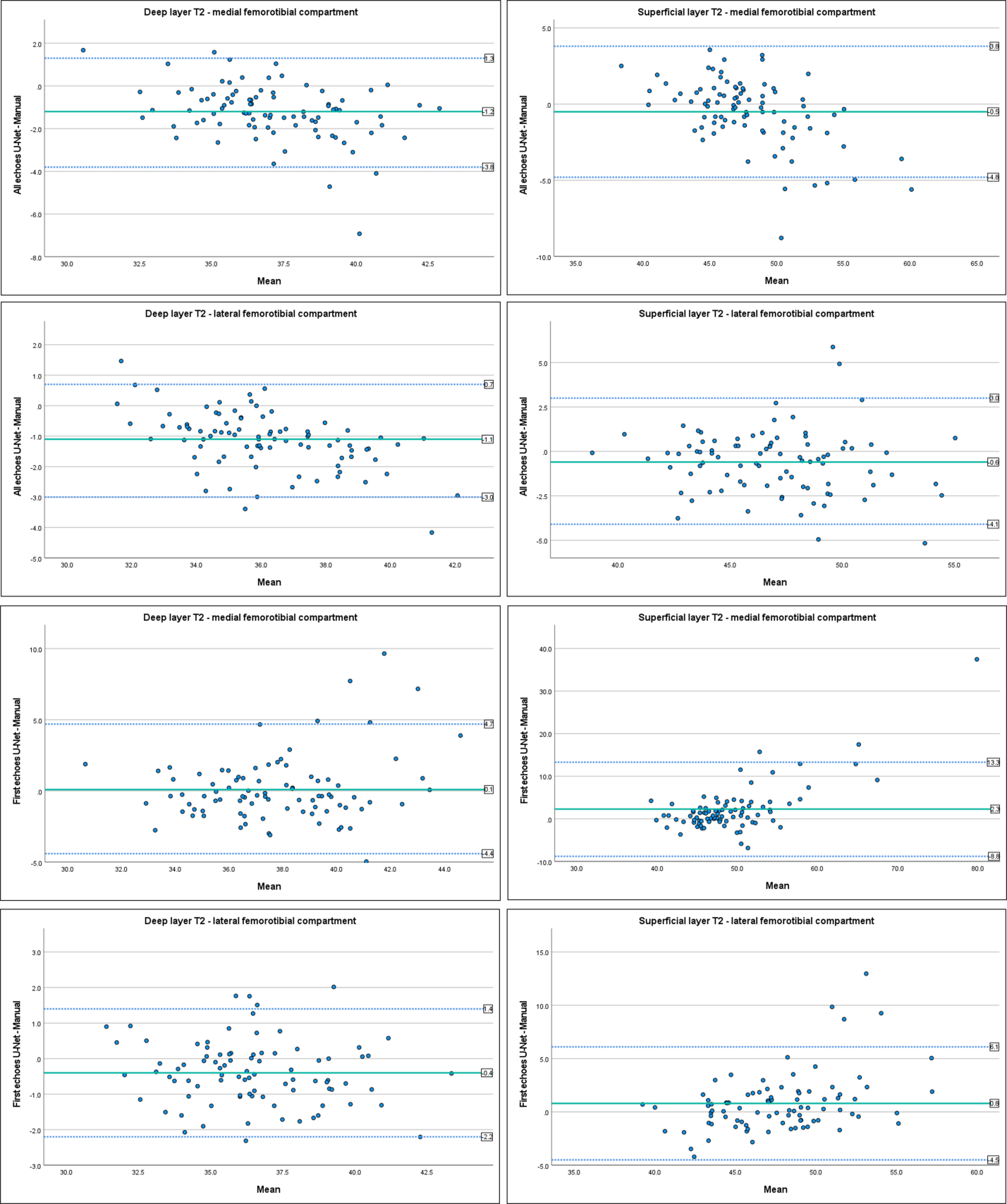

Supplement: Supplementary file 1 — Supplementary file1 (DOCX 1918 KB) [file 256_2024_4786_MOESM1_ESM.docx]
